# African swine fever virus impairs porcine alveolar macrophages bactericidal function by disrupting lysosomal acidification and cathepsin activity

**DOI:** 10.1371/journal.ppat.1014573

**Published:** 2026-09-10

**Authors:** Zhen Xu, Fengyang Shi, Zhiyong Xiang, Runzhi Guo, Zhenyu Wen, Yajin Qu, Quanlin Li, Qiongqiong Zhou, Peng Gao, Yongning Zhang, Xinna Ge, Jun Han, Xin Guo, Lei Zhou, Hanchun Yang

**Affiliations:** 1 State Key Laboratory of Veterinary Public Health Safety, College of Veterinary Medicine, China Agricultural University, Beijing, P.R. China; 2 Key Laboratory of Animal Epidemiology of Ministry of Agriculture and Rural Affairs, College of Veterinary Medicine, China Agricultural University, Beijing, P.R. China; Pirbright Institute, UNITED KINGDOM OF GREAT BRITAIN AND NORTHERN IRELAND

## Abstract

African swine fever virus (ASFV) is a devastating pathogen that poses a severe threat to the global swine industry. ASFV primarily targets the porcine monocyte-macrophage system, which is crucial for defending against bacterial infections via phagocytosis and subsequent intracellular degradation. Clinically, ASFV infection can be complicated by severe secondary bacterial infections. This creates a compelling paradox: while prior *in vitro* studies indicate that ASFV actually increases the phagocytic activity of porcine alveolar macrophages (PAMs), clinical observations frequently report severe secondary bacterial infections. This contradiction led us to hypothesize that the downstream intracellular bactericidal clearance might be compromised. Here, utilizing an *in vitro* co-infection model, it was demonstrated that ASFV significantly impairs the bactericidal capacity of PAMs against representative bacteria (*Escherichia coli*, *Glaesserella parasuis, and Streptococcus suis*), facilitating their intracellular survival and persistence. Although ASFV infection triggers massive reactive oxygen species (ROS) production, this oxidative stress remains functionally ineffective because phagolysosomal acidification and structural integrity are profoundly impaired. Mechanistically, at the late stage of infection, ASFV launches a multipronged assault on the endolysosomal network. Structurally, ASFV inhibits phagosomal and lysosomal acidification while inducing oxidative stress-driven severe lysosomal membrane permeabilization (LMP), leading to reduced phagolysosome volume and physical depletion of the lysosomal pool. Molecularly, ASFV impairs degradative capacity through the transcriptional suppression and blunted lysosomal enrichment of vacuolar (H^+^) ATPase (V-ATPase) subunits, alongside the disruption of lysosomal protease cathepsin D (CTSD) maturation. Furthermore, systematic screening identified six core candidate viral proteins, including CP530R, D129R, E183L, O174L, Q706L, and QP509R, that profoundly suppress both *ATP6V0D* and *CTSK* transcription, further exacerbating this functional impairment. Collectively, our *in vitro* findings reveal that ASFV dismantles host phagolysosomal acidification, thereby neutralizing the microbicidal potential of infected macrophages and potentially converting them into a permissive niche for secondary bacterial pathogens. These observations provide critical new mechanistic insights that may help explain ASFV-associated immune dysfunction at the cellular level.

## Author summary

African swine fever virus (ASFV) is a devastating pathogen for the global swine industry. ASFV primarily infects the porcine mononuclear phagocytes, whereas macrophages eliminate bacteria mainly through phagocytosis and bactericidal activity. Our previous *in vitro* studies have demonstrated that ASFV infection enhances the phagocytic capacity of PAMs. This observation appears paradoxical in light of field reports documenting increased secondary bacterial infections in ASFV-affected herds, and our present findings provide a mechanistic basis for reconciling these seemingly contradictory phenomena at the cellular level. Here, we demonstrate that ASFV significantly impairs the bactericidal capacity of PAMs, facilitating intracellular bacterial survival and delayed clearance. Strikingly, although ASFV infection triggers elevated ROS production, this oxidative burst is rendered functionally ineffective by the profound impairment of phagolysosomal acidification and structural collapse. Mechanistically, late-stage ASFV infection induces cytoplasmic alkalization, halts phagolysosomal acidification, and triggers severe lysosomal membrane permeabilization (LMP). This paralysis is driven by the transcriptional repression and impaired recruitment of V-ATPase to lysosomes upon bacterial challenge, the disrupted maturation of the lysosomal protease cathepsin D, and the targeted transcriptional repression of cathepsin K by specific viral proteins. Collectively, our findings reveal a novel immune suppression strategy whereby ASFV functionally cripples host phagolysosomal acidification and lysosomal protease activity. This provides critical new insights into virus-bacteria synergy and the pathogenesis of secondary infections during ASFV outbreaks.

## Introduction

African swine fever virus (ASFV) is a highly contagious and lethal pathogen that poses a major threat to the global swine industry. Despite its initial discovery as early as 1921 [1], along with its subsequent rapid global spread and ongoing prevalence across multiple continents [2–4], our current understanding of ASFV pathogenic mechanisms remains limited, leaving a great challenge for vaccine development as well as effective prevention and control in the field. ASFV is a complex nucleocytoplasmic large DNA virus (NCLDV) encoding over 160 proteins [5,6], a substantial proportion of which remain functionally uncharacterized or poorly understood [7]. While ASFV infection is typically characterized by high fever, systemic haemorrhaging, and up to 100% mortality [8,9], it concurrently induces rapid and profound immunosuppression that renders the host highly vulnerable to secondary bacterial infections. The exact cellular mechanisms by which virulent ASFV compromises host antimicrobial defenses to allow bacterial persistence represent a critical gap in our understanding of the virus’s pathogenesis.

ASFV primarily targets monocyte-macrophage lineage cells [10], which serve as the primary line of innate immune defense against bacterial invasion, exerting their antimicrobial defenses through the phagocytosis and subsequent killing of pathogens. Our recent studies have demonstrated that ASFV infection of porcine alveolar macrophages (PAMs) enhances their phagocytic capacity for bacteria [11]. This enhanced bacterial uptake presents an intriguing paradox when contrasted with the clinical reality, where ASFV infection is frequently complicated by severe secondary bacterial infections. This contradiction prompted us to hypothesize that although ASFV-infected macrophages can efficiently engulf bacteria, they may fail to effectively kill and degrade the internalized pathogens. Elucidating the mechanisms by which viruses subvert macrophage antimicrobial functions provides a critical framework for understanding the pathogenesis of secondary bacterial infections. For instance, human cytomegalovirus (HCMV), Epstein-Barr virus (EBV), influenza virus, rhinovirus, respiratory syncytial virus (RSV), porcine reproductive and respiratory syndrome virus (PRRSV), and Newcastle disease virus (NDV) have been shown to induce macrophage dysfunction [12–15]. However, these viruses typically suppress both the phagocytic and bactericidal capacities of macrophages. Consequently, whether ASFV specifically suppresses the bactericidal activity of target cells, and the exact molecular mechanisms underlying this process, remain largely unclear.

Macrophages exert their microbicidal functions primarily through the maturation of phagolysosomes [16]. The potent bactericidal activity within these specialized compartments is orchestrated by a synergistic dual-attack system: the rapid generation of reactive oxygen and nitrogen species (ROS/RNS), as well as a strictly regulated non-oxidative armamentarium comprising antimicrobial peptides and hydrolytic enzymes [12,17–19]. The non-oxidative clearance dictates several strict prerequisites, including the successful maturation of phagolysosomes, the maintenance of a highly acidic luminal environment necessary for the activation of acid hydrolases (such as cathepsins, lipases, phosphatases, nucleases, and sulfatases), and the precise sorting and delivery of these enzymes from the trans-Golgi network (TGN) [20]. It has been reported that several viruses disrupt macrophage bactericidal capacity and promote their own replication by regulating cellular metabolism or lysosomal acidification [13–15].

Previous studies have indicated that ASFV infection alters lysosomal morphology, including reduced acidification and decreased organelle size [21]. While ASFV initially relies on the acidic luminal pH of late endosomes to trigger virion disassembly and facilitate the release of its viral core into the cytoplasm, it actively impairs subsequent lysosomal maturation once infection is established [22–28]. ASFV efficiently evades terminal degradation by circumventing the activities of mature lysosomes, including the disruption of autophagosome-lysosome delivery [12]. Furthermore, the virus compromises TGN function, which not only augments viral membrane protein activity and suppresses host immunoregulatory proteins, but also markedly slows the transport of essential proteolytic enzymes.

By exploiting the early endosomal environment for uncoating while simultaneously crippling mature lysosomal acidification and TGN-mediated hydrolytic capacity, ASFV successfully resists degradation by proteolytic enzymes. However, whether this extensive modulation of the endolysosomal environment directly translates to a suppression of the macrophage’s intracellular bactericidal activity remains poorly understood.

To elucidate how ASFV subverts the bactericidal function of macrophages, a comprehensive *in vitro* evaluation model was established. Systematic assessment of both oxidative and non-oxidative antimicrobial mechanisms revealed that ASFV profoundly impairs the intracellular degradation of phagocytosed bacteria. Mechanistically, while viral infection triggers robust oxidative stress, it simultaneously orchestrates a targeted blockade of the endolysosomal pathway. The virus induces cytosolic alkalinization, restricts phagosome and lysosome acidification, diminishes phagolysosome volume, and represses key hydrolytic enzymes. Collectively, these *in vitro* findings demonstrate that ASFV creates a permissive intracellular environment for bacterial survival and persistence in infected macrophages. This provides a mechanistic basis that potentially contributes to the severe secondary infections reported in clinical cases, although further *in vivo* studies are required to validate this hypothesis.

## Results

### ASFV impairs the bactericidal activity of infected macrophages

To investigate the dynamic changes in the bactericidal capacity of ASFV-infected PAMs, a series of fluorescent *Escherichia coli* (*E. coli*) strains (expressing EGFP, mCherry, BFP, or a GFP-mCherry fusion) were engineered for subsequent imaging and quantification (S1A–S1C Fig). Then an *in vitro* macrophage bactericidal evaluation model was established by optimizing the key inoculation parameters, specifically the bacteria-to-macrophage ratio and the internalization period. PAMs were challenged with *E. coli* at various multiplicities of infection (MOIs) and for different co-incubation times. Subsequent quantification of viable intracellular bacteria via agar plate counts, corroborated by fluorescence microscopy, demonstrated that an MOI of 10 combined with a 2-hour co-incubation time yielded an optimal intracellular bacterial load for downstream assays (S1D Fig).

**Fig 1:**
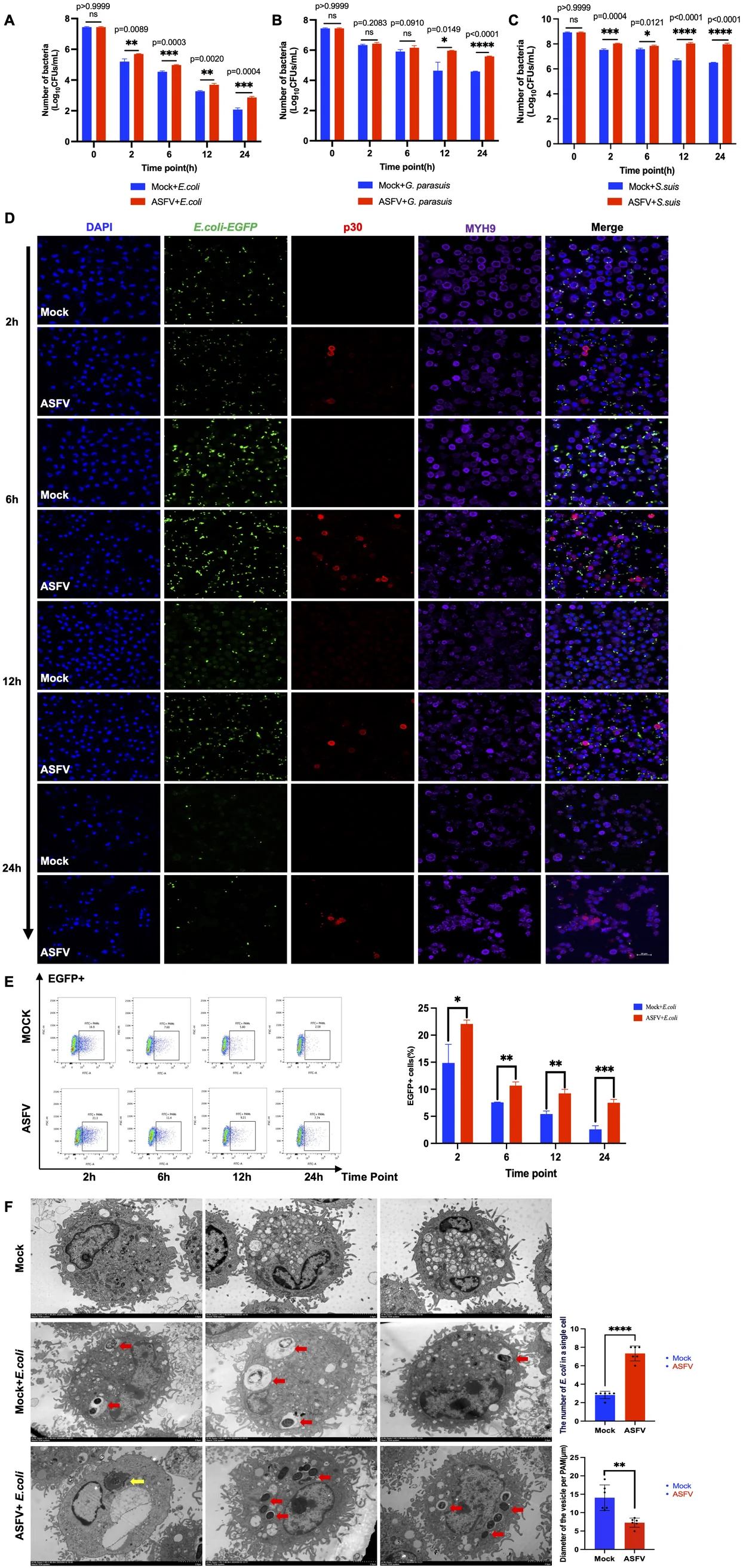
ASFV-infected macrophages exhibit impaired intracellular bactericidal function. A-C. Plate count results of PAMs phagocytizing *E. coli* (MOI 10), *G. parasuis* (MOI 25) or *S. suis* (MOI 10) at various time points following ASFV infection (MOI 0.5) for 24 h. Bacteria were inoculated at the indicated MOI, with 0 h designated as the initial time of infection. Following a 2-hour incubation, gentamicin was applied to eliminate extracellular bacteria, followed by replacement with fresh medium. Plate counting was performed at the indicated time points post-inoculation. D-E. Immunofluorescence assay (IFA) analysis and flow cytometry scatter plots of PAMs phagocytizing *E. coli-EGFP* (MOI 10) at 24 h after ASFV infection (MOI 0.5). Scale bar, 20 µm. Representative data from multiple experiments are shown. F. Electron microscopic results of PAMs infected with ASFV (MOI 0.5) for 24 h, followed by incubation with *E. coli* (MOI 10) for 6 h. The statistical graphs show the number of *E. coli* (upper panel) and the diameter of bacteria-containing vesicles (lower panel) per cell, respectively. Yellow arrows: Virus factories. Red arrows: *E. coli*. Representative data from multiple fields of view is shown. Data were presented as mean ± SD from n = 3 independent experiments. *P* values were calculated by a two-tailed unpaired t-test. *ns*, *P* > 0.05; * *P* < 0.05; ** *P* < 0.01; *** *P* < 0.001; **** *P* < 0.0001.

Building upon the established bactericidal model, plate counting, flow cytometry, and confocal microscopy were employed to assess the impact of ASFV infection on the intracellular bactericidal activity of PAMs. Notably, ASFV infection significantly exacerbated the intracellular bacterial burden across all time points examined, from 2 h to 24 h post-inoculation. This impaired bactericidal capacity was consistently evidenced by higher viable bacterial counts in plating assays (Fig 1A), a greater accumulation of fluorescent *E. coli* visualized by microscopy (Fig 1D), and an elevated percentage of *E. coli*-positive PAMs quantified via flow cytometry (Fig 1E).

Under clinical conditions, *Glaesserella parasuis* (*G. parasuis*) and *Streptococcus suis* (*S. suis*) frequently emerge as major secondary pathogens in cases of viral coinfection [29,30]. To determine whether the impaired macrophage bactericidal phenotype observed with the engineered *E. coli* extends to clinically relevant pathogens, we evaluated clinical isolates of *G. parasuis* and *S. suis* using the identical experimental protocol. For *G. parasuis*, intracellular bacterial load in the ASFV-infected group was initially higher than that in the control group at 2 h and 6 h post-inoculation, though without statistically significant differences; however, the number of surviving bacteria in the infected group remained significantly higher than that in the mock group at 12 h and 24 h post-phagocytosis (Fig 1B). Notably, for *S. suis*, intracellular survival of bacteria in the ASFV-infected group was significantly elevated compared to the control group across all time points examined, from 2 h to 24 h post-inoculation (Fig 1C), indicating a more pronounced and immediate bactericidal defect.

Furthermore, transmission electron microscopy (TEM) analysis revealed pronounced ultrastructural damage in ASFV-infected PAMs. These cells exhibited mitochondrial swelling, severe cytoplasmic vacuolization, and a conspicuous reduction in both the number of lysosomes and the volume of phagosomes. This physical depletion of the lysosomal pool, likely driven by severe membrane damage, suggests a structural collapse of the host’s bactericidal machinery. Notably, a significant accumulation of intracellular bacteria with intact structural morphology was observed within the infected macrophages. These morphological alterations indicate that ASFV infection severely impairs the intracellular degradation of internalized bacteria, which is a direct consequence of both the physical loss of lysosomal vesicles and the functionally compromised phagosomal and lysosomal integrity. Taken together, these data demonstrate that ASFV infection fundamentally disrupts the bactericidal machinery of PAMs (Fig 1F).

### ASFV infection induces oxidative stress in PAMs

Following the recognition and phagocytosis of pathogens, macrophages deploy both oxygen-dependent and oxygen-independent pathways to eliminate engulfed microorganisms within phagolysosomes. To evaluate the impact of ASFV on the oxygen-dependent antimicrobial system, intracellular reactive oxygen species (ROS) levels were first quantified using an H_2_DCFDA probe. When an oxidative environment exists within the cell, this probe is oxidized to green fluorescent DCF, which can be used to detect intracellular ROS levels. Compared to mock-infected controls, ASFV infection induced significantly higher ROS accumulation in both WSL-R4 cells and primary PAMs (S2A and S2B Fig). Furthermore, the generation of reactive nitrogen species (RNS) was also investigated. Inducible nitric oxide synthase (iNOS), a key enzyme activated during infection to catalyze the production of bactericidal nitric oxide (NO), was evaluated. Following ASFV infection of PAMs, although iNOS mRNA transcriptional levels were significantly upregulated at multiple time points, NO secretion levels in the culture supernatant did not show a corresponding significant increase (S2C Fig).

To further characterize the cellular redox state, antioxidant capacity and lipid peroxidation were evaluated. ASFV infection diminished superoxide dismutase (SOD) enzymatic activity in both WSL-R4 cells and PAMs, with significant differences at most time points in PAMs, whereas malondialdehyde (MDA) levels remained unchanged. Interestingly, RT-qPCR analysis revealed a significant upregulation of *SOD* mRNA in ASFV-infected PAMs, likely reflecting a compensatory transcriptional response to the depleted antioxidant enzyme pool. The transcription of other redox-regulatory genes, including *HO-1* and *NQO1*, also exhibited a non-significant upward trend (S2D–S2F Fig). Western blot analysis was subsequently performed to validate the expression of oxidative stress-related proteins in PAMs at various time points following ASFV infection. The results demonstrated that ASFV infection suppressed the Keap1-Nrf2-HO-1 pathway and concurrently downregulated the expression levels of antioxidant proteins Catalase, GPX1, and GPX4 (S2G Fig).

Given that mitochondria are primary sources of intracellular ROS, mitochondrial superoxide production was specifically monitored using MitoSOX Red staining. This probe specifically targets mitochondria and produces intense red fluorescence upon oxidation by superoxide. The analysis demonstrated a marked accumulation of mitochondrial superoxide in ASFV-infected PAMs (S3A–S3C Fig). Excessive oxidative stress typically leads to mitochondrial dysfunction; therefore, JC-1 dye was used to assess mitochondrial integrity. Normal mitochondria emit intense red fluorescence, whereas green fluorescence is produced when mitochondrial membrane potential is low. Consistent with the elevated ROS levels, ASFV infection induced a pronounced decrease in mitochondrial membrane potential, indicative of severe mitochondrial damage (S3D Fig).

### ASFV disrupts intracellular pH homeostasis and lysosomal proteolytic function

**Fig 2:**
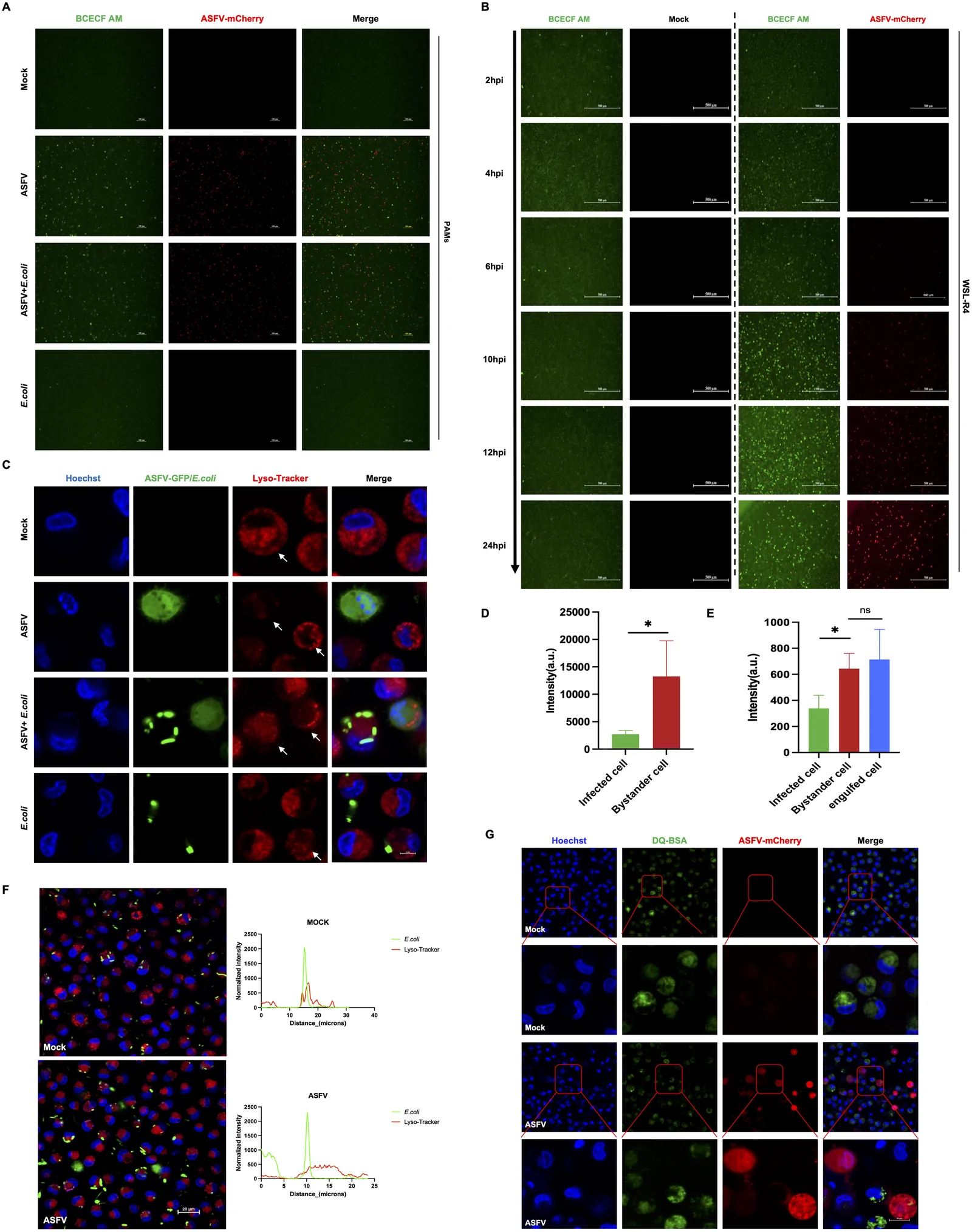
ASFV infection disrupts intracellular pH homeostasis in PAMs. A. Cytoplasmic pH in PAMs infected with ASFV (MOI 0.5) for 24 hpi followed by *E. coli* inoculation (MOI 10) for 6 h. Scale bar, 100 µm. B. Cytoplasmic pH in WSL-R4 cells infected with ASFV-mCherry (MOI 0.5) at different time points. Scale bar, 500 µm. C. Lysosomal acidification levels in PAMs infected with ASFV-GFP (MOI 0.5) for 24 hpi followed by *E. coli* inoculation (MOI 10) for 6 h. White arrows: Acidified lysosomes. Scale bar, 5 µm. D-E. Analysis of the fluorescence intensity of lysosome-specific staining in the infected group (infected cells and bystander cells) and the post-infection co-culture group (infected cells, bystander cells, and *E. coli*-phagocytosing cells) of PAMs infected with ASFV (MOI 0.5) for 24 hpi followed by *E. coli* inoculation (MOI 10) for 6 h. F. Lysosome-*E. coli* colocalization levels in PAMs infected with ASFV-GFP (MOI 0.5) for 24 hpi followed by *E. coli* inoculation (MOI 10) for 6 h. Scale bar, 20 µm. G. DQ-BSA biohydrolysis capacity in PAMs infected with ASFV-mCherry (MOI 0.5) for 24 hpi. Scale bar, 10 µm. Representative data from multiple fields of view is shown. Data were presented as mean ± SD from n = 3 independent experiments. *P* values were calculated by a two-tailed unpaired t-test. *ns*, *P* > 0.05; * *P* < 0.05.

Although ASFV infection induces robust oxidative stress, it paradoxically impairs the overall bactericidal activity of macrophages. To reconcile this apparent paradox, we investigated the impact of ASFV on oxygen-independent clearance mechanisms, with particular emphasis on intracellular pH dynamics. Cytosolic pH was monitored using the cell-permeant probe BCECF-AM, which forms green fluorescence at appropriate pH values, and the intensity of green fluorescence increases with increasing pH. ASFV-mCherry infection induced a significant alkalinization of the cytosol in PAMs, whereas *E. coli* challenge or mock treatment did not elicit such changes (Fig 2A). Similarly, ASFV-mCherry-infected WSL-R4 cells exhibited a marked elevation in cytosolic pH. Time-course analysis revealed a progressive rise in cytosolic pH following viral challenge, which became significantly elevated by 10 hpi (Fig 2B). To further assess lysosomal function, Lyso-Tracker Red fluorescent probe, which is highly specific for acidic organelles, was used to label lysosomes in red. Lysosomal acidification was markedly impaired in ASFV-GFP-infected PAMs compared to controls. Notably, this defect was restricted to virus-infected cells, as adjacent non-infected bystander cells maintained normal acidification levels, and exposure to *E. coli* alone had no significant effect on lysosomal pH (Fig 2C–2E). Subsequent examination of the co-localization between lysosomes and EGFP-expressing *E. coli* via live-cell imaging at 24 hpi revealed a pronounced reduction in the overlap between bacteria and lysosomes in virus-positive macrophages. This indicates that ASFV infection fundamentally disrupts intracellular pH homeostasis, impairing the acidification of the compartments harboring internalized bacteria (Fig 2F).

Lysosomes are enriched with diverse acid hydrolases—including approximately 60 kinds of pH-dependent proteases, lipases, and nucleases—that require a highly acidic luminal environment for activation to degrade pathogen cell walls and other components. By elevating cytosolic pH and simultaneously suppressing lysosomal acidification, ASFV compromises the catalytic activity of these catabolic enzymes. To directly assess whether ASFV infection functionally impairs lysosomal proteolytic capacity, PAMs pre-infected with ASFV-mCherry for 24 h were loaded with DQ-BSA Green, a self-quenched fluorescent substrate that emits intense green fluorescence only upon proteolytic cleavage. Live-cell imaging revealed bright fluorescent signals in control cells, indicative of robust proteolytic degradation of the BSA substrate. In stark contrast, ASFV-infected macrophages exhibited a substantially dimmer fluorescence signal, demonstrating that the substrate remained largely intact (Fig 2G). Therefore, while viral infection triggers substantial oxidative stress, the profound impairment of macrophage bactericidal function is primarily driven by ASFV-induced pH dysregulation and subsequent lysosomal failure.

### ASFV infection induces a late-stage defect in phagosomal acidification

**Fig 3:**
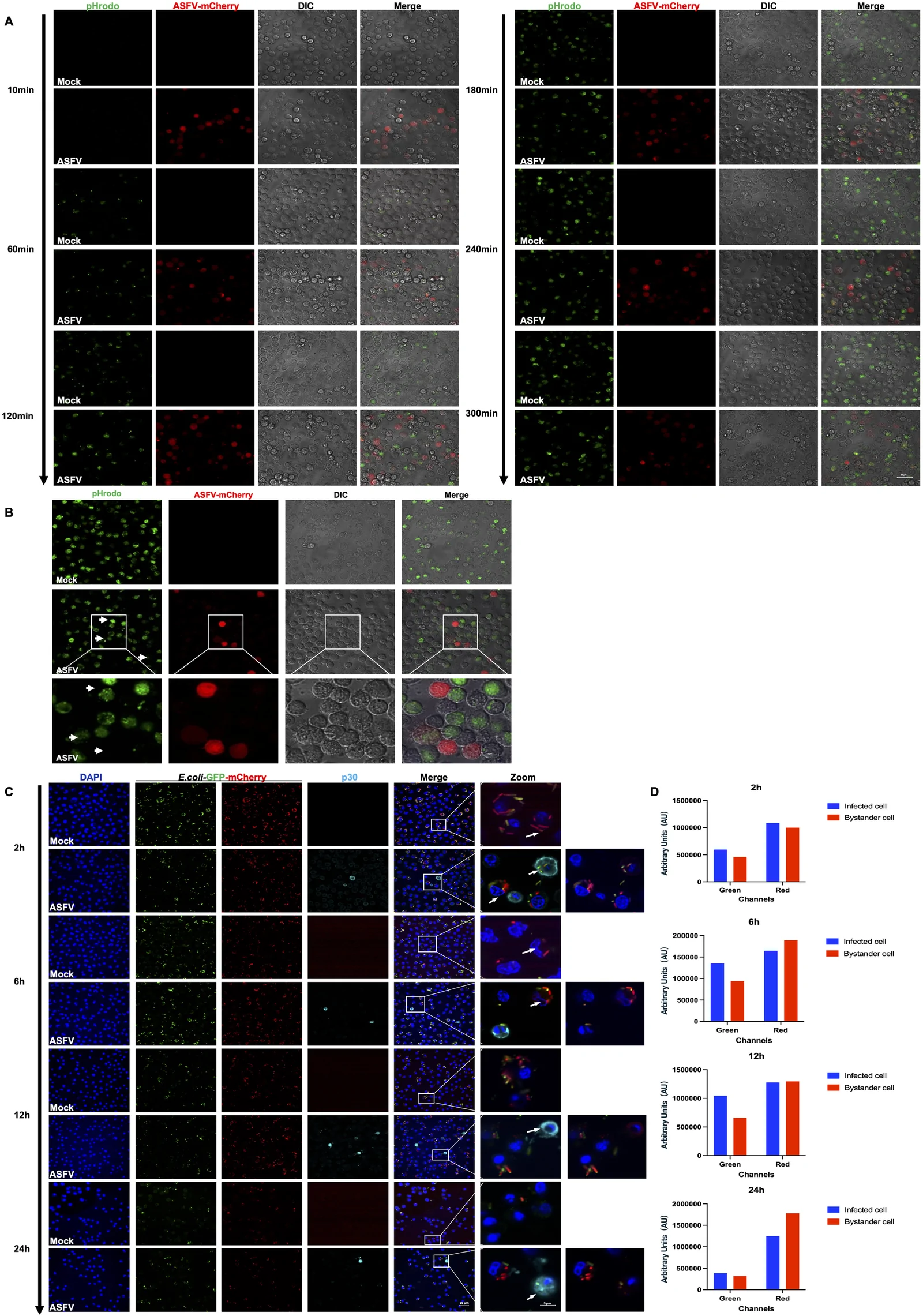
ASFV infection inhibits phagosomal acidification in PAMs. A. Live-cell imaging of phagocytosis of pHrodo-conjugated *E. coli* (MOI 10) by PAMs pre-infected with ASFV-mCherry (MOI 0.5) for 24 h. B. Live-cell imaging of phagocytosis (6 h) of pHrodo-conjugated *E. coli* (MOI 10) by PAMs pre-infected with ASFV-mCherry (MOI 0.5) for 24 h. Thick white arrows: pHrodo-labeled *E. coli* in phagosomes of infected cells. Scale bar, 10 µm. C. PAMs were infected with ASFV (MOI 0.5) for 24 hpi, subsequently inoculated with *E. coli*-GFP-mCherry (MOI 10), and the quenching profiles of GFP and mCherry fluorescence were monitored and quantified at designated time points post-inoculation. Thin white arrow: *E. coli*-GFP-mCherry in uninfected and infected cells. Scale bar, 20 µm.

Following bacterial engulfment, macrophages form phagosomes that mature into microbicidal phagolysosomes via acidification and lysosomal fusion. Given that ASFV infection elevates cytosolic pH and inhibits lysosomal acidification, it was hypothesized that phagosomal acidification might be similarly disrupted. To evaluate this, PAMs pre-infected with ASFV-mCherry for 24 h were challenged with pHrodo Green-conjugated *E. coli*, a pH-sensitive indicator that exhibits bright fluorescence exclusively in acidic environments. Live-cell imaging revealed that while pHrodo-labeled bacteria underwent progressive acidification in mock-infected controls (beginning approximately 60 min post-phagocytosis), this fluorescence signal was markedly attenuated or completely absent in ASFV-infected macrophages, denoting severe acidification impairment. Notably, adjacent uninfected bystander cells maintained normal acidification dynamics (Fig 3A and 3B).

To comprehensively determine whether ASFV concurrently disrupts acidification within both phagosomes and lysosomes, an *E. coli* strain co-expressing GFP and mCherry was engineered. In this dual-reporter system, the acid-sensitive GFP is quenched within acidic phagolysosomes, whereas mCherry fluorescence remains stable. Consequently, successful acidification yields a solely red signal, while defective acidification presents as a yellow (GFP + mCherry) signal. Following a 24-h pre-infection with wild-type ASFV, PAMs were challenged with these dual-fluorescent bacteria and monitored via live-cell imaging. Compared to the robust GFP quenching observed in controls, ASFV-infected cells retained strong, unquenched GFP fluorescence and harbored elevated bacterial loads. These data further validate that ASFV infection effectively blocks endolysosomal acidification, thereby arresting phagolysosomal maturation (Fig 3C and 3D).

**Fig 4:**
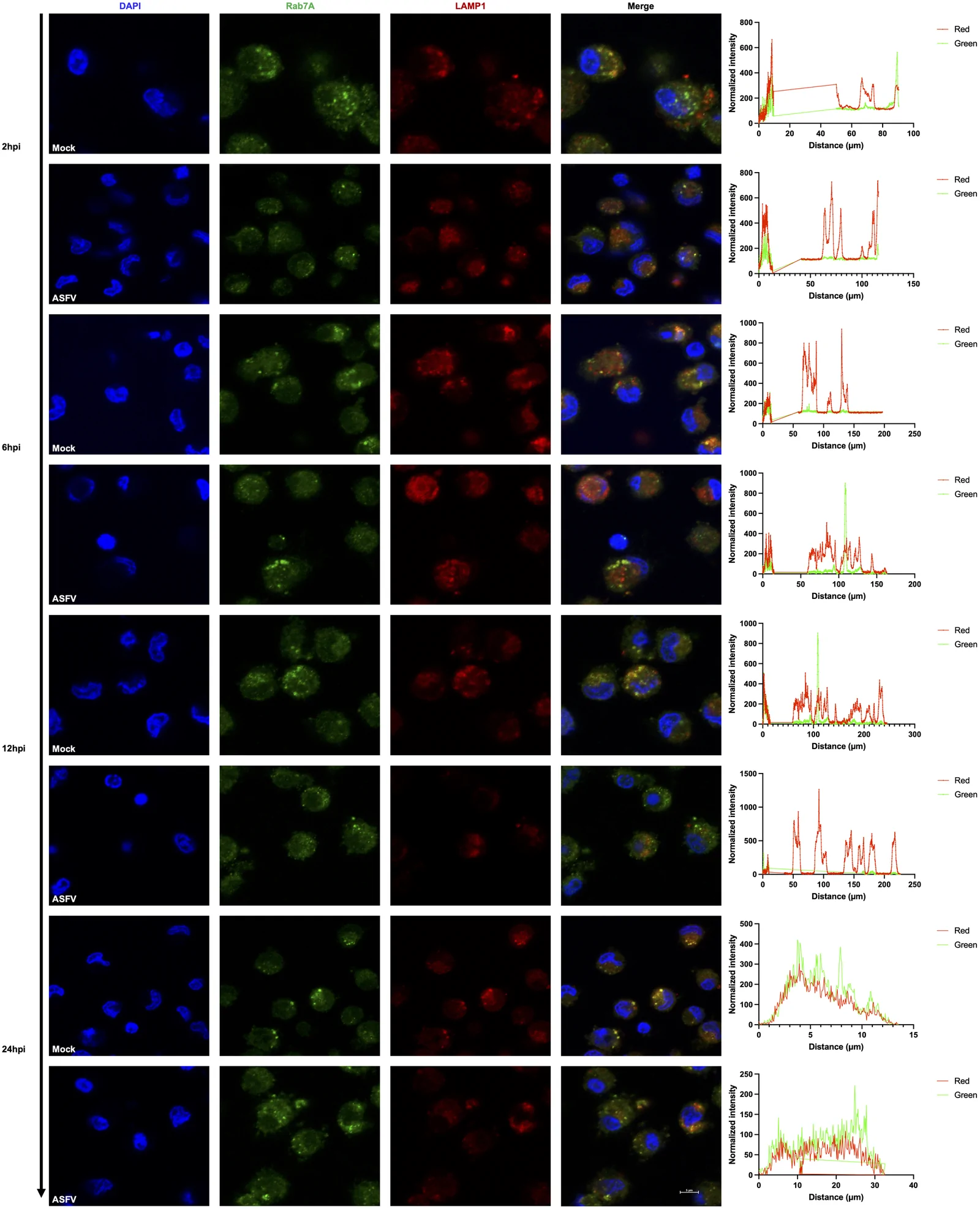
Late-phase ASFV infection in PAMs suppresses endolysosomal acidification. Co-localization analysis of late endosomal Rab7A and lysosomal membrane protein LAMP1 in PAMs infected with ASFV (MOI 0.5) for 24 h. Rab7-to-LAMP1 transition reflects general endolysosomal maturation. Scale bar, 5 µm.

Subsequent investigations assessed the impact of ASFV on endolysosomal trafficking and maturation. Through a time-course tracking assay utilizing Rab7 (a late endosome marker) and LAMP1 (a lysosomal marker), the dynamics of vesicular convergence onto the degradative compartment were monitored. Colocalization analysis indicated that while ASFV infection does not impede Rab7-to-LAMP1 transition during the early stages, it significantly disrupts this maturation sequence at later time points (Fig 4).

The colocalization of *E. coli-EGFP* with LAMP1, LAMP2, CTSD, and ATP6V0D was subsequently analyzed, revealing that ASFV infection of PAMs significantly diminished colocalization levels across all markers. Furthermore, the temporal colocalization dynamics between latex beads and Lyso-Tracker after 24 h of ASFV infection were assessed, demonstrating that late-stage ASFV infection attenuated the colocalization of latex beads with LysoTracker over time (S4 Fig).

Taken together, these findings demonstrate that ASFV infection profoundly disrupts intracellular pH homeostasis and inhibits phagosomal acidification at the late-stage, while simultaneously impairing downstream endo-lysosomal trafficking. This extensive perturbation of the endolysosomal network provides a clear mechanistic basis for the virus-induced paralysis of macrophage bactericidal activity.

### ASFV infection remodels the lysosomal proteome and suppresses functional acidification pathways

**Fig 5:**
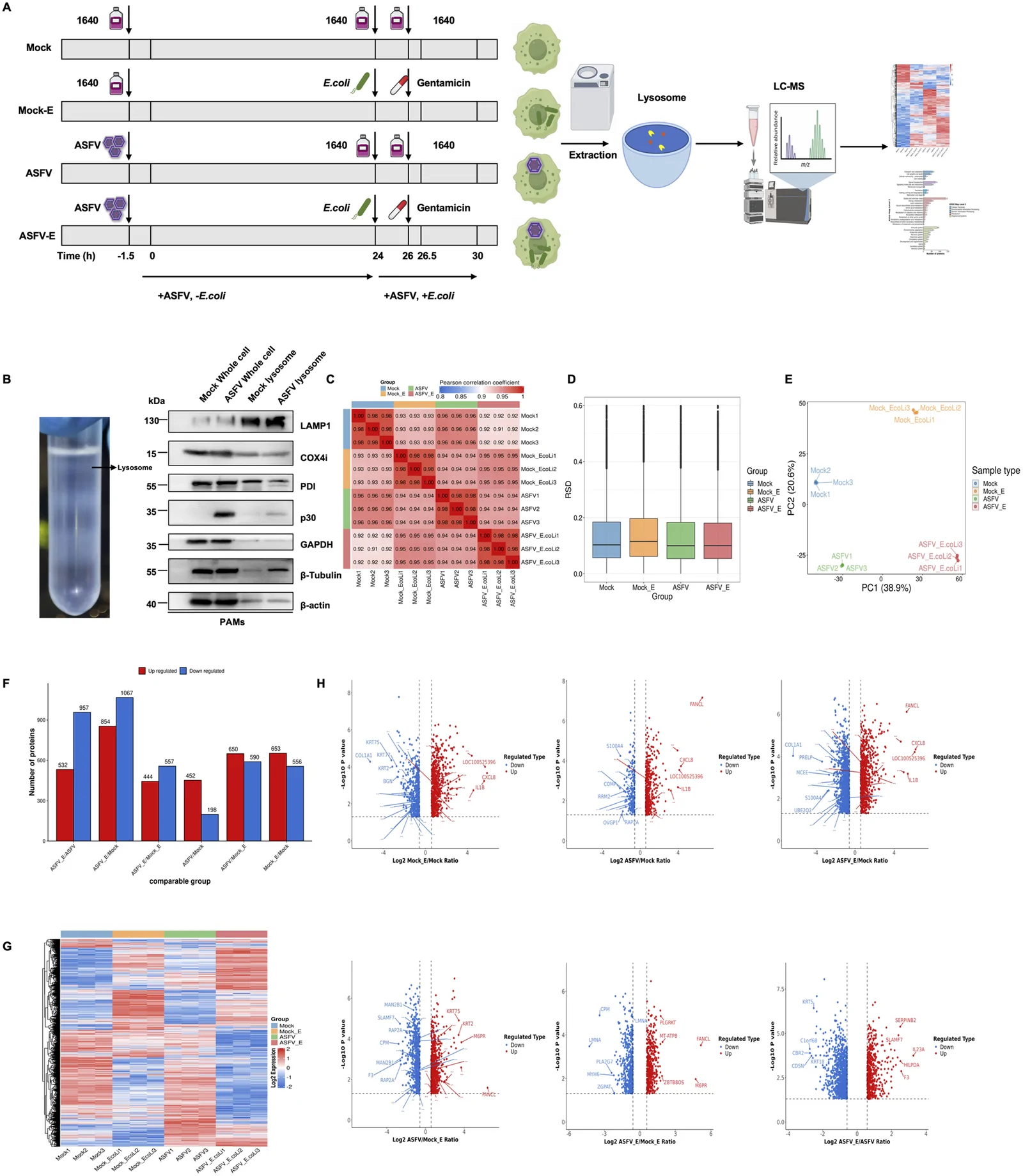
4D-SmartDIA proteomic quantification of lysosomes in ASFV-infected PAMs. A. Pattern diagram of lysosomal isolation, purification and proteomic analysis. PAMs were treated with different methods, and cells were collected at designated time points for lysosome isolation, trypsin digestion, and analysis on the timsTOF LC-MS (liquid chromatography-mass spectrometry) platform. B. Lysosomal enrichment and purification by ultracentrifugation, with the top fraction containing the isolated and purified lysosomes. The purity and efficiency of lysosomal isolation from uninfected and ASFV-infected PAMs were assessed by Western blot. C-E. Pearson’s Correlation Coefficient (PCC), Relative Standard Deviation (RSD), Principal-component analysis (PCA) of Mock, Mock-E, ASFV and ASFV-E samples. F-H. Statistical maps, heat map and volcano plots of differentially expressed proteins among the four lysosomal sample groups. Differential protein expression was determined by calculating the fold change (FC) as the ratio of mean relative quantification values between two groups, with statistical significance assessed by two-tailed Student’s t-test on log2-transformed data (*P* < 0.05); proteins with FC > 1.5 or FC < 1/1.5 were considered significantly upregulated or downregulated, respectively.

To investigate how ASFV infection weakens lysosomal bactericidal function, 4D-SmartDIA quantitative proteomic analysis was performed on enriched lysosomes derived from four experimental groups of PAMs: untreated (Mock), *E. coli*-challenged (Mock-E), ASFV-infected (ASFV), and ASFV-infected followed by *E. coli* challenge (ASFV-E) (Fig 5A–5E). A total of 39,218 host peptides, representing 6,151 host proteins, along with 27 ASFV proteins, were identified.

Consistent with previous reports [31,32], ASFV infection significantly altered the macrophage proteome. The ASFV-E group exhibited the most pronounced proteomic shifts compared to the Mock group (854 up-regulated and 1,067 down-regulated proteins), indicating that viral-bacterial co-exposure drives profound cellular reprogramming (Fig 5F and 5G). Global functional enrichment analyses (COG, KEGG, and GO) revealed that while general viral responses—such as shifts in energy metabolism and membrane trafficking—were prevalent, the most critical alterations were concentrated within the endolysosomal system. Specifically, pathways related to phagosome maturation, lysosome organization, and antigen processing were markedly dysregulated following ASFV infection (S5–S9 Figs).

Given the essential role of lysosomes in pathogen clearance, subsequent analysis was focused on the proteomic shifts between the ASFV-E and Mock-E groups to evaluate degradative capacity. While *E. coli* challenge alone (Mock-E) upregulated numerous inflammatory mediators and transmembrane transporters, prior ASFV infection (ASFV-E) robustly suppressed the macrophage hydrolytic machinery. Specifically, 557 proteins were significantly downregulated in the ASFV-E group compared to the Mock-E group. This downregulated cluster prominently featured a broad spectrum of lysosomal acid hydrolases (including multiple cathepsins and nucleases). Concurrently, KEGG analysis confirmed that while Toll-like receptors were upregulated on phagosomes, the downstream lysosomal expression of acidic hydrolases and membrane integrins was severely suppressed. This suggests a critical viral-induced dissociation of initial pathogen recognition from effective intracellular degradation.

The optimal catalytic activity of lysosomal hydrolases is strictly dependent on an acidic microenvironment, which is primarily maintained by the V-ATPase proton pump and associated ion channels. From the proteomic dataset, 114 core pH-regulating proteins were identified and classified into the V-ATPase family (24 members), chloride channels (34 members), Na⁺ /H⁺ exchangers (14 members), and other ion transporters (42 members).

We focused on the V0 domain of the V-ATPase complex, which is responsible for pumping protons from the cytoplasm into lysosomes to maintain an acidic luminal environment, as well as the cathepsins that mediate the proteolytic degradation of engulfed bacteria within lysosomes. Notably, V-ATPase subunits exhibited the most pivotal dynamic alterations. At the protein level, two detected V0 domain subunits, along with multiple acid hydrolases, were robustly upregulated in the Mock-E group, reflecting a normal bacteria-induced acidification response. However, prior ASFV infection effectively subverted this defense mechanism. Compared to the Mock-E group, the ASFV-E group displayed significantly reduced expression of V0 domain subunits and acid hydrolases, indicating a viral-mediated reversal of the bacteria-induced acidification response. Interestingly, ASFV infection alone strongly upregulated the mannose-6-phosphate receptor (M6PR), potentially altering the trafficking of cathepsins to the lysosome.

**Fig 6:**
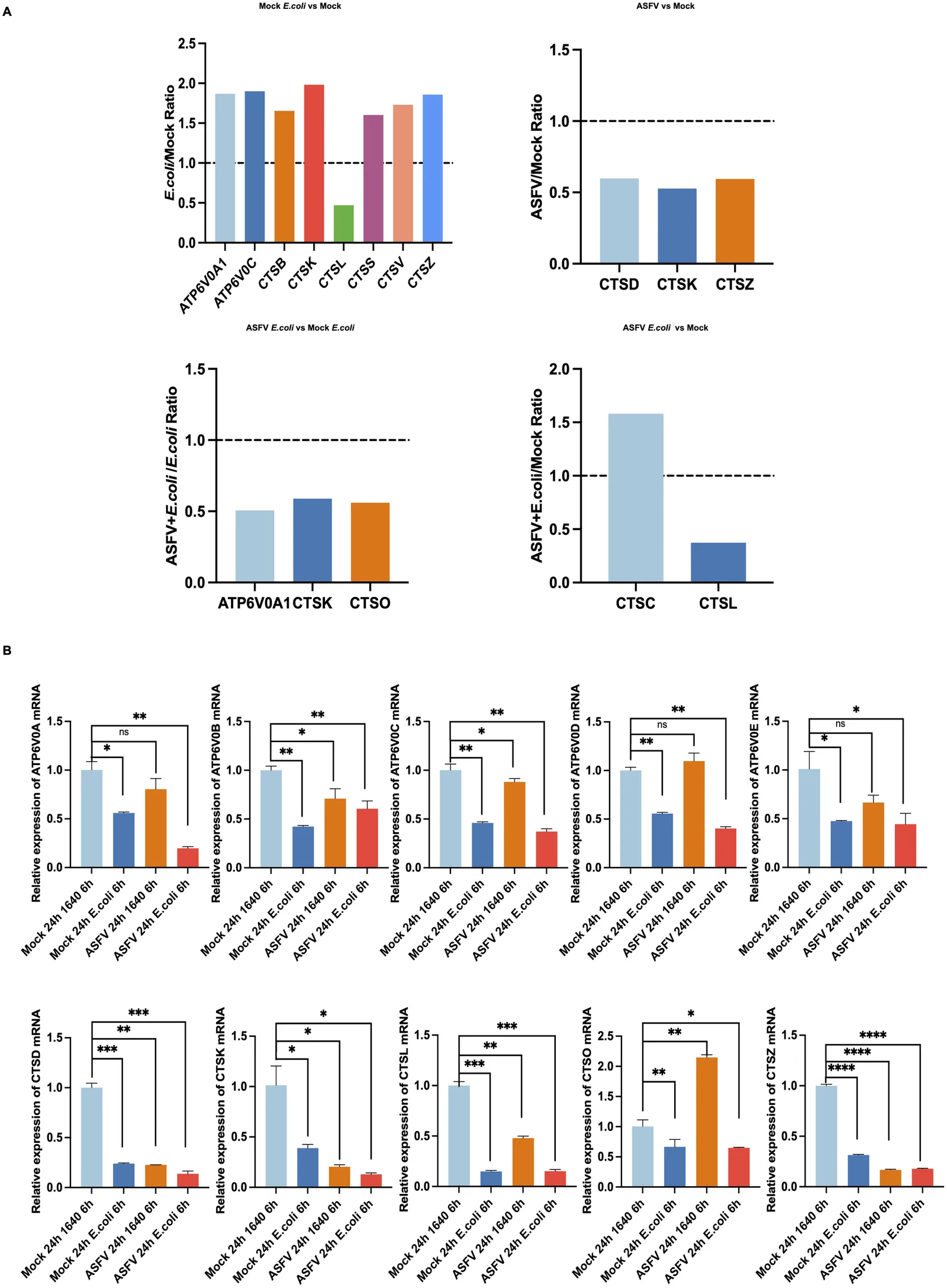
Transcriptional and expression analysis of pH-related proteins among differentially expressed lysosomal proteins. A. Quantitative proteomic comparison of V-ATPase proton pumps and acid hydrolases in lysosomes of Mock, Mock-E, ASFV, and ASFV-E groups. Differential protein expression was determined by calculating the fold change (FC) as the ratio of mean relative quantification values between two groups, with statistical significance assessed by two-tailed Student’s t-test on log2-transformed data (*P* < 0.05); proteins with FC > 1.5 or FC < 1/1.5 were considered significantly upregulated or downregulated, respectively. B. RT-qPCR analysis of transcriptional changes in different subunits of V-ATPase proton pumps, acid hydrolases, and lysosomal membrane proteins at the whole-cell level in Mock, Mock-E, ASFV, and ASFV-E samples. Data were presented as mean ± SD from n = 3 independent experiments. *P* values were calculated by a two-tailed unpaired t-test. ns, *P* > 0.05; * *P* < 0.05; ** *P* < 0.01; *** *P* < 0.001; **** *P* < 0.0001.

To elucidate the relationship between lysosomal protein abundance and whole-cell gene transcription, 4D-SmartDIA proteomic data were compared with RT-qPCR results (Fig 6). In response to *E. coli* challenge alone (Mock *E. coli* vs Mock), a distinct divergence between transcription and protein enrichment occurred: the lysosomal protein levels of V-ATPase subunits (e.g., ATP6V0A1 and ATP6V0C) and acid hydrolases (e.g., CTSK and CTSZ, but not CTSL) were upregulated, while their corresponding whole-cell mRNA levels decreased, implying a potential post-transcriptional translocation or stabilization mechanism that warrants further validation. Conversely, ASFV infection alone (ASFV vs Mock) concurrently suppressed both the lysosomal protein accumulation and whole-cell mRNA transcription of specific key acid hydrolases, namely CTSD, CTSK, and CTSZ. Under viral-bacterial co-challenge (ASFV *E. coli* vs Mock *E. coli*), ASFV specifically repressed the lysosomal protein accumulation of several V-ATPase subunits (ATP6V0A1) and hydrolases (CTSK and CTSO). Despite gene-specific transcriptional variations—such as the marked upregulation of CTSO mRNA during isolated ASFV infection—the overall protein enrichment of critical acidification and hydrolytic components in lysosomes remained functionally suppressed by the virus.

Collectively, these findings raise the possibility that ASFV facilitates secondary bacterial infections by targeting the V-ATPase complex and lysosomal acid hydrolases, a hypothesis that requires additional experimental confirmation.

### ASFV infection induces lysosomal membrane permeabilization in PAMs

Lysosomal membrane integrity is essential for maintaining cellular homeostasis. Oxidative damage or endoplasmic reticulum stress can induce lysosomal membrane permeabilization (LMP), leading to hydrolase leakage, luminal alkalinization, and accelerated proton efflux, which may ultimately trigger cell death. To evaluate whether ASFV infection compromises lysosomal membrane integrity in PAMs, acridine orange (AO) uptake and redistribution assays, a well-established method for detecting LMP [33–36], were performed. Fluorescence analysis revealed that ASFV infection caused a significant reduction in intracellular red fluorescence, indicating increased LMP and structural damage to the lysosomes. Notably, this effect became progressively more pronounced with prolonged infection times. Additionally, lactate dehydrogenase (LDH) release was significantly elevated post-infection, suggesting concomitant damage to the plasma membrane. Taken together, these ultrastructural and membrane permeability alterations reflect severe structural damage to the lysosomes.

The concurrent LMP and elevated cytosolic pH indicate a profound, multidimensional dysregulation of cellular ion homeostasis and endolysosomal integrity orchestrated by ASFV. Notably, passive proton leakage from damaged lysosomes typically causes canonical cytosolic acidification; the paradoxical alkalinization observed here prompted us to further dissect the underlying mechanisms. To determine the specific role of ASFV-induced oxidative stress in this dysregulation, we utilized the potent ROS scavenger N-acetyl-L-cysteine (NAC). First, assessing the general impact of ROS on the viral life cycle, we found that NAC treatment significantly inhibited ASFV replication (S11A Fig). Next, we evaluated the contribution of ROS to lysosomal structural damage. Acridine orange staining demonstrated that NAC-mediated ROS clearance significantly alleviated ASFV-induced LMP (S10 Fig), establishing oxidative stress as a primary driver of membrane permeabilization. However, alleviating this structural damage did not translate to functional restoration. Cytosolic pH measurements revealed that NAC treatment failed to alleviate ASFV-induced cytosolic alkalinization (S11B Fig), indicating that pH dysregulation occurs independently of ROS accumulation. Concurrently, in ASFV-bacterial co-infection models, NAC supplementation could not rescue the impaired bactericidal capacity of PAMs (S11C Fig). These results confirm that while ROS drives physical lysosomal damage, the functional failure in bacterial clearance is dictated by an independent disruption of phagolysosomal pH homeostasis.

### Interference with pH-related proteins inhibits macrophage bactericidal function

To functionally validate the importance of the acidification machinery identified in the proteomic analysis, siRNA-mediated knockdown of key V-ATPase subunits was performed. Knockdown efficiency was verified, with siRNAs achieving approximately 80% silencing in WSL-R4 cells and 50% in primary PAMs (S12A Fig). Due to the technical challenges of transfecting primary PAMs, initial screening was performed in WSL-R4 cells, with key findings validated in primary PAMs. Subsequent evaluation using the BCECF-AM revealed that silencing V-ATPase subunits (siATP6V0A and siATP6V0E) elevated cytosolic pH, phenocopying the alkalinization observed in ASFV-infected cells (S12B Fig), as manifested by enhanced green fluorescence. This confirms that V-ATPase is a critical determinant of phagosomal and lysosomal pH homeostasis. Notably, acute depletion of V-ATPase prior to infection markedly reduced ASFV-GFP replication (S12C Fig), indicating that early endosomal acidification is essential for viral entry and uncoating. This fundamentally contrasts with the late-phase suppression of V-ATPase induced by ASFV, which occurs after replication is established and serves to impair bactericidal function rather than block viral egress. Although the exact mechanism remains unclear, this phenomenon may reflect lysosomal stress responses or a compensatory dysregulation of ion transport pathways.

**Fig 7:**
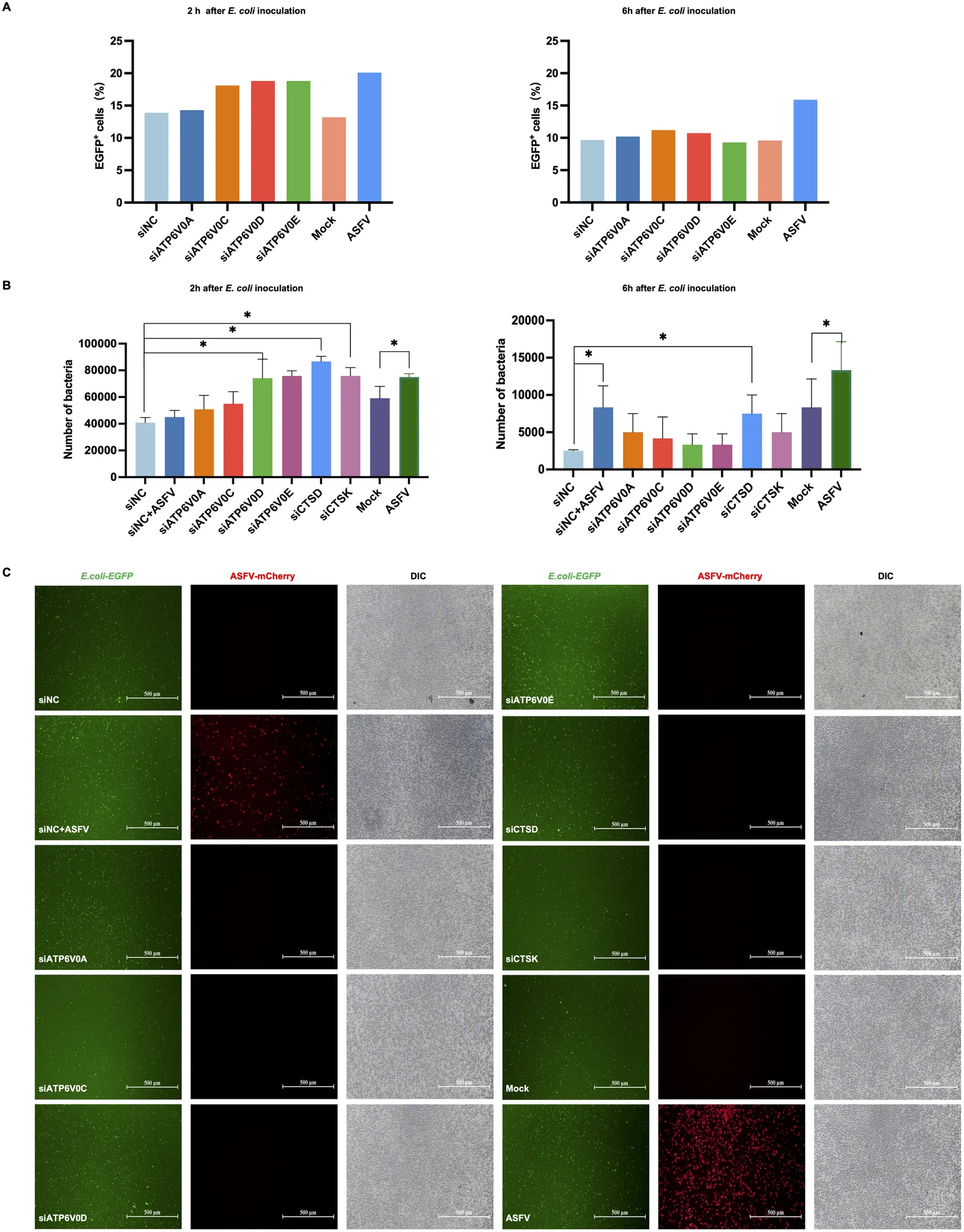
Inhibition of bacterial killing in PAMs after knockdown of pH-related proteins. A-B. Flow-cytometric enumeration of fluorescent-bacteria-positive PAMs and plate-count quantification of viable intracellular bacteria in PAMs 2 h and 6 h after *E. coli* (MOI 10) challenge, performed 24 h post siRNA transfection. C. Fluorescence-microscopy images of intracellular fluorescent bacteria in PAMs 6 h after *E. coli* (MOI 10) challenge, 24 h post siRNA transfection. Scale bar, 500 µm. Data were presented as mean ± SD from n = 3 independent experiments. *P* values were calculated by a two-tailed unpaired t-test. *ns*, *P* > 0.05; * *P* < 0.05.

Bactericidal capacity was subsequently quantified via flow cytometry and colony counting. At 2 h post-challenge with *E. coli*-EGFP, the percentage of fluorescent bacteria-positive cells was higher across all knockdown groups compared to siNC controls, although it remained lower than that observed in ASFV-infected cells. Correspondingly, intracellular viable bacterial counts were elevated in the knockdown groups—most notably with siATP6V0E, siCTSD, and siCTSK. While the siNC group did not differ from uninfected controls, ASFV infection alone yielded a significantly higher viable bacterial burden. By 6 h post-bacterial challenge, this pattern persisted: silenced groups contained more fluorescent bacteria and exhibited higher viable counts than both the siNC and mock groups, yet remained below the peak levels seen with ASFV infection. Fluorescence microscopy at 6 h post-challenge corroborated these results; ASFV infection markedly increased the abundance of intracellular fluorescent bacteria. Among the knockdown conditions, cells treated with siATP6V0A, siATP6V0E, siLAMP1, and siCTSD displayed the highest bacterial loads (Fig 7). Finally, confocal imaging of lysosomal acidification demonstrated impaired acidification across all siRNA-treated cells, with siATP6V0D and siATP6V0E producing the most profound inhibition (S13 Fig).

Taken together, these data conclusively demonstrate that the depletion of V-ATPase subunits or key acid hydrolases is sufficient to compromise lysosomal acidification and significantly impair the bactericidal capacity of PAMs. This directly phenocopies the defects observed during ASFV infection, suggesting that the virus-induced downregulation of these specific targets is a primary molecular driver of macrophage bactericidal failure, functioning in tandem with the aforementioned structural collapse.

### Genome-wide screening identifies ASFV proteins regulating cellular pH and cathepsin transcription

**Fig 8:**
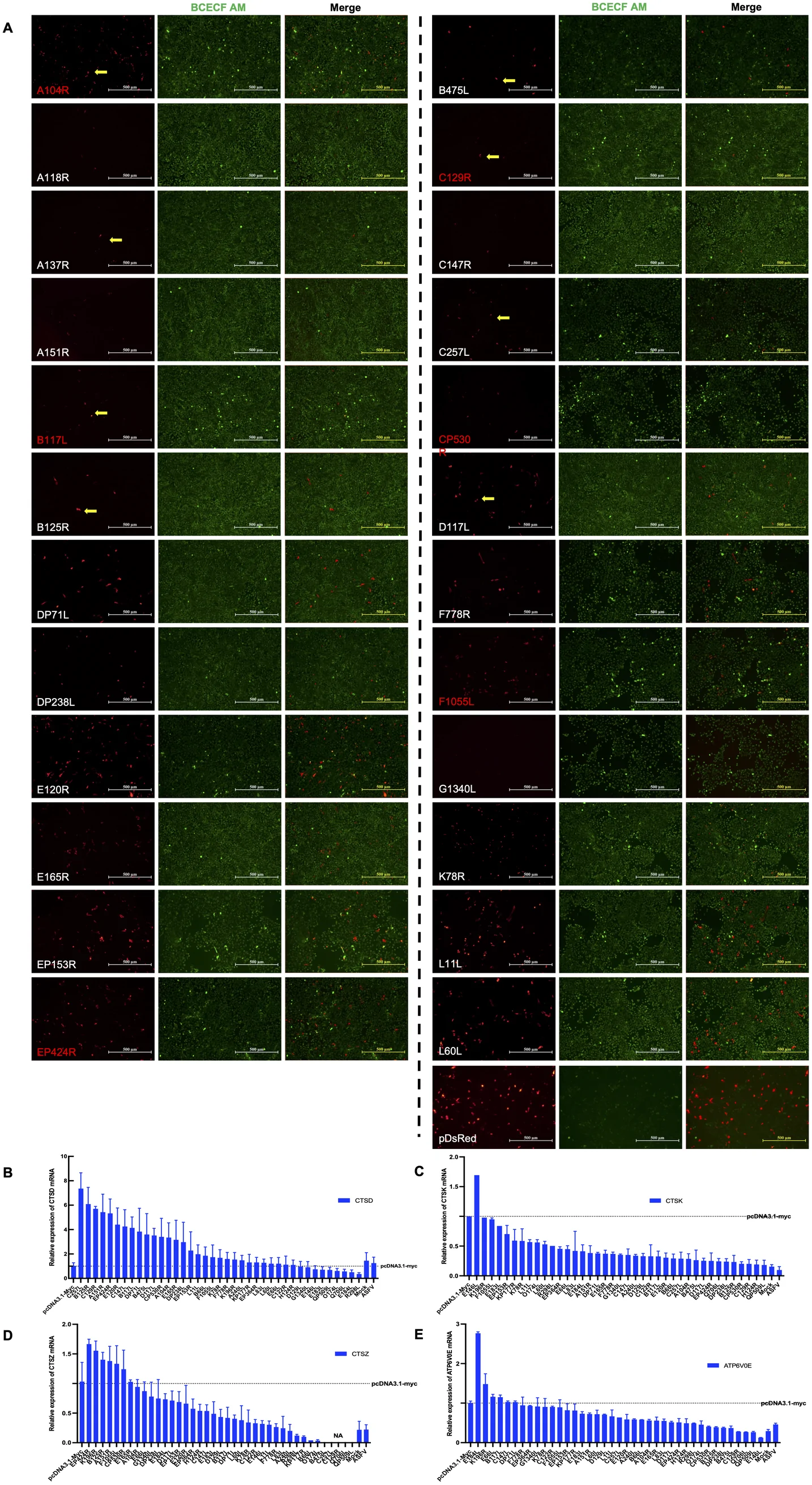
Screening of viral proteins that raise cytosolic pH. A. WSL-R4 cells were transfected with ASFV protein-encoding plasmids fused to red fluorescent protein, and cytosolic pH was measured using BCECF-AM. Scale bar, 500 µm. B-E. Screening of viral proteins that suppress transcription of pH-related genes. WSL-R4 cells were transfected with individual ASFV protein-encoding plasmids for 24 h, followed by RT-qPCR analysis of mRNA levels of pH-related genes.

To identify ASFV-encoded proteins that modulate lysosomal acidification, WSL-R4 cells were initially transfected with a library of 136 codon-optimized ASFV open reading frames (ORFs), and their effects on cytosolic pH were systematically measured. From this primary screen, 24 candidates that induced pronounced pH shifts were selected for further validation. Each candidate was cloned in-frame with a red fluorescent protein into the pDsRed2 vector to enable expression visualization. These 24 RFP-tagged constructs were subsequently reintroduced into WSL-R4 cells and rescreened for their ability to alter cytosolic pH. While expression levels varied among the constructs—ranging from high to undetectable—several well-expressed proteins, specifically A104R, B117L, C129R, CP530R, EP424R, F1055L, and L11L, consistently elevated cytosolic pH (Fig 8A).

Building upon these initial observations, the secondary transcriptional screen was expanded to include a total of 39 viral proteins (comprising the initial 24 candidates plus an additional 15 ORFs from the library that also induced notable pH shifts). These 39 selected viral proteins were ectopically expressed in WSL-R4 cells, and their subsequent impact on the transcription of V-type ATPase subunits and lysosomal acid hydrolases was quantified via RT-qPCR. Interestingly, while the majority of the tested viral proteins upregulated *CTSD* mRNA levels, they concurrently downregulated the transcripts of *CTSK*, *CTSZ*, and *ATP6V0E.* Additionally, nine specific constructs successfully reduced *CTSD* mRNA expression. Notably, six core candidate viral proteins—including CP530R, D129R, E183L, O174L, Q706L, QP509R, and R298L—profoundly suppressed both *ATP6V0D* and *CTSK* transcription, yet, contrary to the general trend, did not diminish *CTSZ* transcript levels (Fig 8B–8E). Through this comprehensive screening approach, six core candidate proteins (CP530R, D129R, E183L, QP509R, O174L, Q706L) were ultimately identified from the 136 ASFV ORFs as potent regulators of macrophage acidification. However, the precise molecular mechanisms by which these viral effectors regulate intracellular pH and lysosomal function warrant further in-depth investigation.

## Discussion

Clinical ASFV infections are characterized by profound immunosuppression and severe secondary bacterial infections, which significantly complicate disease control and prevention. While it is established that ASFV extensively manipulates host innate immunity, the precise mechanisms by which it paralyzes macrophage bactericidal function remain obscure. This study provides compelling evidence that ASFV dismantles the phagolysosomal axis via a bipartite mechanism that combines structural collapse with transcriptional silencing, leading to impaired bactericidal activity. Using an *in vitro* macrophage co-infection model, we observed that although ASFV infection triggers robust ROS production, it impairs bactericidal capacity not by inhibiting this oxidative burst. Instead, the virus triggers lysosomal membrane permeabilization (LMP), which physically depletes the available lysosomal pool, while simultaneously suppressing V-ATPase-mediated acidification and globally reprogramming the lysosomal proteome. These findings suggest that ASFV-infected macrophages may become permissive environments for prolonged bacterial survival and persistence, providing a mechanistic basis for understanding secondary bacterial infections reported in field studies. Instead of a specific viral interference with the canonical oxidative burst, our data suggest that the massive ROS accumulation is largely a pathological consequence of ASFV-induced mitochondrial damage. Although ROS generation by NADPH oxidase is not strictly contingent on phagosomal acidification, efficient pathogen killing relies on the coordinated action of ROS and lysosomal acid hydrolases [37–39]. The deacidification of phagolysosomes in ASFV-infected cells disrupts this coordination. While the oxidative burst remains robust, the failure to activate acid-dependent enzymes renders the ROS functionally impotent against bacteria. Instead of achieving microbial clearance, this excessive and misdirected oxidative stress promotes organelle damage and macrophage death [40,41]. Thus, ASFV-induced pH elevation effectively creates a permissive intracellular niche by dissociating ROS production from the downstream enzymatic antimicrobial machinery. Moreover, NAC-mediated ROS scavenging experiments further established a causal relationship between ASFV-induced ROS accumulation and lysosomal membrane permeabilization, confirming that ROS-driven LMP constitutes a critical mechanism for the physical disruption of lysosomal integrity. Importantly, this structural damage operates in parallel with V-ATPase-mediated deacidification, and together they act synergistically to orchestrate the complete paralysis of macrophage bactericidal function.

**Fig 9:**
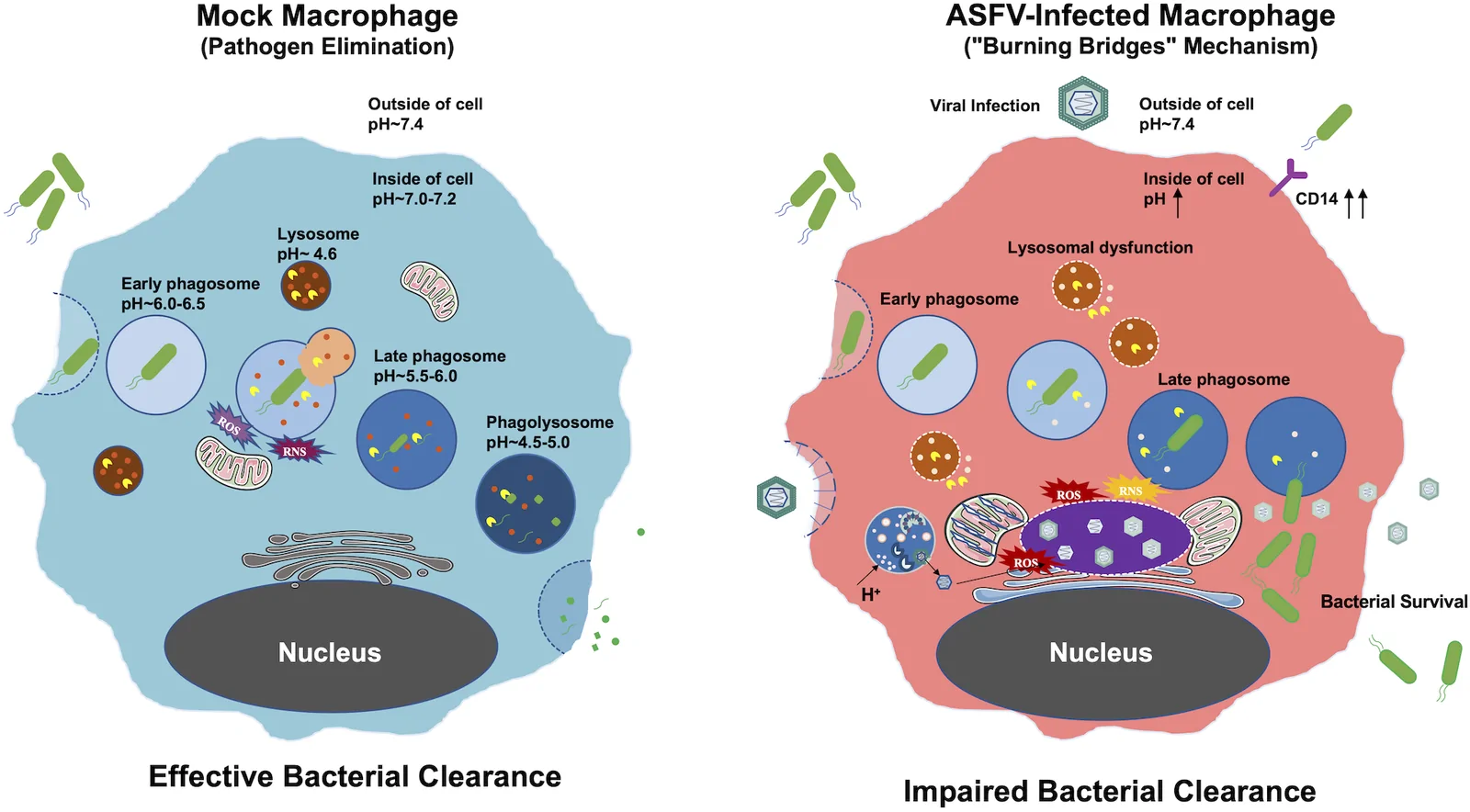
Model diagram of ASFV infection inhibiting the bactericidal function of PAMs. In the late stage of ASFV infection, intracellular oxidative stress and cytoplasmic alkalinization are induced, inhibiting the acidification of phagosomes and lysosomes in infected cells, increasing lysosomal membrane permeability and reducing hydrolytic activity, while downregulating the expression of proton pumps and acidic hydrolases, ultimately leading to impaired bactericidal function of PAMs.

Integration of proteomic, qPCR, and Western blot data revealed a complex, layer-specific regulation of lysosomal components in ASFV-infected macrophages. In PAMs stimulated with *E. coli* alone, lysosomal proteomics showed upregulation of V-ATPase subunits and cathepsins at the protein level, whereas qPCR analysis of whole-cell mRNA revealed concurrent transcriptional downregulation of these same genes. This discordance suggests that the early response to bacterial challenge involves rapid post-transcriptional recruitment or stabilization of pre-existing lysosomal proteins, despite a concomitant reduction in de novo transcription. ASFV strategically exploits and exacerbates this endolysosomal failure through divergent manipulation of key hydrolases. The data demonstrate that viral infection selectively suppresses cathepsin transcription and blunts the bacteria-induced lysosomal enrichment of V-ATPase subunits. However, we acknowledge that the present data, while informative, do not fully dissect the relative contribution of each layer, and further investigation using subunit-specific functional assays is warranted. Furthermore, a paradoxical upregulation of M6PR was observed alongside a sharp decline in mature CTSD at the protein level. It is hypothesized that M6PR upregulation is a futile compensatory response by the host cell attempting to rescue lysosomal function. Given that ASFV is known to disrupt TGN trafficking [20,42], combined with the neutralized lysosomal pH observed in this study, pro-CTSD delivery and proteolytic cleavage into its active form are significantly impaired. In stark contrast to CTSD’s post-translational blockade, the depletion of CTSK is driven directly at the transcriptional level. Our systematic screening revealed that specific late-stage viral proteins, such as CP530R and D129R, potently repress *CTSK* and *ATP6V0D* mRNA expression. This devastating combination of translational/trafficking blockade (CTSD) and targeted transcriptional repression (CTSK) leads to a profound and comprehensive collapse of the non-oxidative killing mechanism (Fig 9).

Modulating endolysosomal pH is a conserved strategy among intracellular pathogens, albeit with diverse tactics. While RNA viruses like SARS-CoV-2 and influenza A virus deacidify lysosomes primarily to facilitate viral egress or uncoating, the present findings suggest that ASFV utilizes deacidification to prolong host cell survival, while simultaneously shutting down immune sensing. Instead of achieving a persistent state, the extensively infected macrophages, driven by unmitigated oxidative stress and severe lysosomal membrane permeabilization, ultimately release LDH and show ultrastructural damage consistent with lytic death, releasing massive amounts of cytokines (e.g., TNF-α, IL-1β) that contribute to the hemorrhagic fever and systemic inflammation observed *in vivo* [32,43].

To accurately reflect the clinical reality of swine farms, a co-infection model of primary PAMs with *G. parasuis* or *S. suis*, two highly prevalent secondary pathogens in ASFV outbreaks, was used. While this *in vitro* model effectively recapitulates the cell-autonomous defects in macrophage phagolysosomal function, it is acknowledged that it cannot fully capture the complex, systemic immune interactions, such as inter-organ cytokine crosstalk and neutrophil recruitment, that occur in a living host. Future *in vivo* studies using attenuated ASFV strains or specific viral protein deletion mutants are warranted to validate these findings within the tissue microenvironment. Importantly, the functional screening identified several ASFV proteins, notably CP530R and D129R, capable of directly perturbing cellular pH when ectopically expressed. However, transient overexpression outside the context of a highly coordinated viral replication cycle can occasionally yield non-physiological artifacts. Therefore, elucidating the exact roles of these viral effectors necessitates future validation using recombinant gene-deleted ASFV mutants. Elucidating the precise molecular interactions between these viral effectors and host ion channels will be the focus of subsequent investigations, offering promising new targets for the rational design of antiviral therapeutics and live-attenuated vaccines.

Due to the difficulty of achieving ideal genetic manipulation in primary cells, the low and unstable efficiency of siRNA interference, the technical challenges of protein overexpression, and the lack of an immortalized cell line with both phagocytic function and ASFV susceptibility, these compounding technical bottlenecks significantly complicated the mechanistic validation in primary cells. Although WSL-R4 cells lack phagocytic function, we still used them for many experiments corresponding to those with PAMs, aiming to determine whether the cellular effects are common and to facilitate corroboration or substitution of PAM data during transfection and interference experiments. Therefore, finding more efficient methods for transfecting primary PAMs or constructing usable passaged cell lines both represent challenges that need to be addressed in future research.

Finally, we acknowledge a technical limitation: all bactericidal assays were performed with ASFV at an MOI of 0.5, meaning only a fraction of target cells were directly infected at the time of bacterial challenge. We therefore cannot fully rule out the possibility that the observed bactericidal effects partly reflect bystander responses from uninfected cells rather than a direct consequence of infection. To address this, we attempted to increase the effective MOI by virus ultrafiltration concentration; however, under higher multiplicities, the extensive cytopathic effect and cell detachment during the late phase of ASFV infection (which is the focus of our study) severely compromised cell monolayer integrity, making it unfeasible to maintain viable, adherent cells for subsequent bacterial co-incubation. Given these practical constraints, we were unable to perform the ideal dose-escalation experiment within the current framework. We recognize this as a clear caveat, and we interpret our data with due caution, while believing that the consistent trends across multiple assays still provide biologically meaningful insights that warrant further investigation under improved infection protocols.

In conclusion, the present work uncovers a fundamental mechanism by which ASFV orchestrates profound macrophage dysfunction to drive concurrent bacterial infections. The data strongly demonstrate that ASFV paralyzes bacterial clearance not by suppressing oxidative bursts, but by redirecting ROS to damage lysosomal membrane integrity, resulting in impaired acidification and lysosomal proteostasis, thereby functionally dissociating ROS production from pathogen degradation. Consequently, infected macrophages are hijacked and converted from competent immune sentinels into non-acidic, highly permissive niches for secondary pathogens like *G. parasuis*. Furthermore, the discovery of viral pH-modulators such as CP530R and D129R bridges a critical gap in understanding ASFV-host interactions. These mechanistic insights significantly advance the current understanding of the severe immunosuppression defining clinical ASFV infections, offering a strategic framework for developing next-generation antiviral interventions and rationally attenuated vaccine candidates.

## Materials and methods

### Ethics statement and biosafety

Preparation of PAMs derived from 30-day-old SPF pigs was performed as described previously [44], under the approval of the Laboratory Animal Welfare and Animal Experimental Ethical Committee of China Agricultural University (Approval No. AW72903202-1-2). All experiments involving live ASFV were performed in the Biosafety Level 3 (BSL-3) Lab at China Agricultural University (license number: 2022-ASFV-002).

### Bacterial strains, cells and viruses

*Glaesserella parasuis* (*G. parasuis*), *Streptococcus suis* (*S. suis*), and *Escherichia coli* (*E. coli*) were prepared in our laboratory. The EGFP, mCherry, BFP, and mCherry-GFP genes were amplified from pEGFP-N2, pCMV-C-mCherry, pCMV-C-BFP, and pCMV-mCherry-GFP-LC3B, respectively, using primers containing *Eco*RI and *Hin*dIII restriction sites. The amplified fragments were then cloned into plasmid pET-28a (+). The recombinant plasmids were transformed into *E. coli* DH5α competent cells for sequencing verification. The confirmed plasmids were subsequently transformed into *E. coli* BL21(DE3) competent cells to construct the *E. coli-EGFP* and *E. coli-mCherry* strains for further use. *Glaesserella parasuis* (*G. parasuis*) serotype 13 was isolated from lung tissue of a deceased pig and streaked onto TSA plates supplemented with 1% NAD and 5% serum. Suspected colonies were transferred to TSB medium containing serum and NAD for successive passages to achieve purity. *Streptococcus suis* (*S. suis*) serotype 2 was isolated from lung and liver samples of deceased pigs and streaked onto THB agar plates containing polymyxin. Suspected colonies were transferred to THB broth supplemented with 10% serum for successive passages to achieve purity. Purified strains were verified by PCR and 16S rRNA gene sequencing, and authenticated isolates were deposited in our laboratory collection. PAMs were obtained using bronchoalveolar lavage as described previously [45]. The cloned wild boar lung (WSL-R4) cell line that supports efficient replication of ASFV was obtained through four rounds of subcloning screening in our laboratory. Both PAMs and WSL-R4 were cultured in RPMI-1640 medium, containing 10% fetal bovine serum (FBS) and penicillin (1000 U/mL) and streptomycin (100 μg/mL) at 37°C with 5% CO_2_. The ASFV CADC_HN09 strain (GenBank accession no: MZ614662.1) was provided by the China Animal Disease Control Center (Beijing, China). The fluorescently labeled recombinant viruses ASFV-GFP and ASFV-mCherry were constructed by deleting the *MGF360-18R* gene and *EP402R* gene from the ASFV-HN09 genome, respectively, and replacing them with GFP and mCherry sequences. The viral titers after propagation in PAMs reached 10^6.5^ TCID_50_/mL. For infection assays, PAMs and WSL-R4 were cultured in RPMI-1640 medium supplemented with 2% FBS, penicillin and streptomycin.

### Antibodies and reagents

The murine monoclonal antibody against ASFV p30 protein (mouse anti-p30 MAb) was prepared by our laboratory. H_2_DCFDA (D399), MitoSOX Red (M36008), Acridine Orange (A1301), PHRODO IFL GREEN STP (P36013), DAPI (62248), Lipofectamine RNAiMAX reagent (13778150), Lipofectamine LTX Reagent with PLUS Reagent (15338100), MYH9 (PA5-29673) rabbit polyclonal antibody, Alexa Fluor 568-conjugated goat anti-mouse IgG antibody (A11019), Alexa Fluor 488-conjugated goat anti-mouse IgG antibody (A11017), Alexa Fluor 488-conjugated goat anti-rabbit IgG antibody (A11070), Alexa Fluor 568-conjugated goat anti-rabbit IgG antibody (A11011), and Alexa Fluor 647-conjugated goat anti-mouse IgG antibody (A21235) were all obtained from Thermo Fisher Scientific. Rab7 (9367T) Rabbit Monoclonal Antibody was purchased from Cell Signaling Technology (Boston, MA, USA). Mouse anti Pig CD107a monoclonal antibody (MCA2315GA) was purchased from Bio-Rad. Fluorescein (FITC)-conjugated goat anti-swine IgG (H + L) (114-095-003) was purchased from Jackson ImmunoResearch. Protease inhibitor cocktail (P8340) was obtained from Sigma-Aldrich. Nitric Oxide Assay Kit (S0021S), Total Superoxide Dismutase Assay Kit with WST-8 (S0101S), LDH Cytotoxicity Assay Kit (C0017), Lipid Peroxidation MDA Assay Kit (S0131M), Lyso-Tracker Red (C1046), and BCECF AM (S1006) were purchased from Beyotime Biotechnology. Antibody dilution buffer (KTZ-001) and Hoechst 33342 (C1029) were purchased from Solarbio Life Sciences. GPX1 Polyclonal antibody (29329-1), GPX4 Polyclonal antibody (14432-1-AP), Catalase Polyclonal antibody (21260-1-AP), SOD1 Polyclonal antibody (10269-1-AP), SOD2 Polyclonal antibody (24127-1-AP), KEAP1 Polyclonal antibody (10503-2-AP), NRF2/NFE2L2 Polyclonal antibody (16396-1-AP), HO-1/HMOX1 Monoclonal antibody (66743-1-Ig), CD107b/LAMP2 Monoclonal antibody (66301-1-Ig), ATP6V0D1 Monoclonal antibody (68506-1-Ig), PDI, COXIV Polyclonal antibody (11242-1-AP), Cathepsin D Polyclonal antibody (21327-1-AP), TFEB Polyclonal antibody (13372-1-AP), β-actin Monoclonal antibody (66009-1-Ig), and β-Tubulin Polyclonal antibody (10094-1-AP) were purchased from Proteintech. Lysosome Enrichment Kit (BB-3603) was purchased from Bestbio. X-tremeGENE HP DNA transfection reagent was purchased from Roche, XTGHP-RO. Enzyme-linked Immunosorbent Assay Kit for Tumor Necrosis Factor Alpha (TNF-α) (SEA133Po) was obtained from Cloud-Clone Corp.

### Viral infection and virus titration

PAMs and WSL-R4 were infected with ASFV at an MOI of 1. After 1.5 h of virus adsorption, the cells were washed thrice with PBS and then replaced with fresh RPMI-1640 media with 2% FBS. At 72 h post-infection, the cells together with supernatants were freeze-thawed thrice. After removal of cell debris by centrifugation, the titer of viral samples was determined on PAMs using a microtitration infectivity assay and the results were interpreted by an indirect immunofluorescence assay based on p30 antibody staining [45]. The viral titers were reported as 50% tissue culture infective dose per milliliter (TCID_50_/mL) according to the Reed-Muench method.

### Bacterial culture and growth curve determination

To establish the relationship between optical density and bacterial viability, *E. coli* was inoculated into TSB medium and monitored hourly for OD_600nm_ values. Concurrently, bacterial suspensions underwent serial ten-fold dilutions and were plated on agar for Colony Forming Unit (CFU) counting. This standardized OD_600nm_-to-CFU calibration curve was subsequently used to adjust bacterial concentrations for all infection assays, including those involving clinical *G. parasuis* isolates. *G. parasuis* were grown overnight at 37°C in TSB medium supplemented with 10 μg/ml nicotinamide adenine dinucleotide (NAD).

### Optimization of the bactericidal model

PAMs were seeded in 24-well plates (1 × 10^6^ cells/well) and allowed to adhere in 10% FBS-supplemented RPMI-1640. Optimization of the bacterial challenge was performed by varying the Multiplicity of Infection (MOI: 1, 10, 20, 40, 60, 80, 100) and incubation times (0.5 h, 1 h, 2 h, 3 h, 4 h). Following co-incubation, extracellular bacteria were eliminated using 100 μg/mL gentamicin for 30 min. Cells were then lysed with 0.2% Triton X-100, and intracellular bacterial loads were quantified via plate counting to determine the optimal conditions (MOI 10, 2 h incubation) for subsequent assays.

### Intracellular bactericidal activity assay

To assess the impact of ASFV on macrophage function, PAMs were infected with ASFV (MOI 0.5) for 24 h prior to bacterial challenge. Infected cells were incubated with *E. coli* (MOI 10), *G. parasuis* (MOI 25), or *S. suis* (MOI 10) for 2 h, followed by gentamicin treatment to remove extracellular pathogens. At 2 h, 6 h, 12 h, and 24 h post-bacterial infection, cells were washed rigorously with PBS and lysed with 0.2% Triton X-100. The released intracellular bacteria were then titrated by serial dilution and plate counting to calculate the time-dependent killing efficiency of the macrophages.

### Immunofluorescence

The immunofluorescence analysis procedures have been described elsewhere [46]. Briefly, PAMs seeded on coverslips in 24-well plates were infected with ASFV at an MOI of 0.1, or 0.5. At indicated time points post infection, the cells were fixed with 3.7% paraformaldehyde for 15 min at room temperature, permeabilized with PBS containing 0.1% Triton X-100 for 10 min, and then blocked with 2% bovine serum albumin (BSA)-PBS for 30 min. The cells were then incubated with primary antibodies as indicated in a humid chamber for 2 h at room temperature or overnight at 4°C. After being washed 3 times with PBS for 5 min each, the cells were incubated with appropriate secondary antibody (Alexa 488, 647 or 568-conjugated) for an additional 1 h. Nuclear DNA was stained with 4’,6-diamidino-2-phenylindole (DAPI) (Thermo Fisher, 62248) for 5 min and then washed with PBS three times for 5 min each. The slides were mounted with Aqua-Poly (Polysciences, 18606–20), and fluorescence was visualized using Nikon A1 confocal microscopy (Nikon Instruments Inc., Tokyo, Japan).

### Confocal immunofluorescence assay

In the infection experiments, PAMs and WSL-R4 grown to ~50% confluence on coverslips in 24-well plates were infected with ASFV-HN09 at an MOI of 0.5 for the specified times post-infection. The cells were stained with Lyso-Tracker Red for 15 min at 37°C before fixation. After 1-h fixation with 3.7% paraformaldehyde and 30-min blocking with 2% BSA, the cells were first probed with the relevant primary antibodies, and then incubated with the corresponding secondary antibodies as described previously [47]. After counterstaining cell nuclei with DAPI, the cells were visualized using a Nikon A1 confocal microscope (Nikon Instruments Inc., Tokyo, Japan).

### Measurement of oxidative stress status

MitoSOX Red Detection Assay Kit (Invitrogen, M36009), H_2_DCFDA (Invitrogen, D399), Nitric Oxide Assay Kit (Beyotime, S0021S), Total Superoxide Dismutase Assay Kit with WST-8 (Beyotime, S0101S), Lipid Peroxidation MDA Assay Kit (Beyotime, S0131S) were used according to manufacturer’s instructions.

### Evaluation of intracellular pH changes

An overnight culture of *E. coli* was labeled with pHrodo-green (Invitrogen, P36013) and resuspended in RPMI-1640 supplemented with 10% FBS. For live-cell imaging, PAMs were incubated with *E. coli* followed by ASFV-GFP/mCherry infection (MOI 0.5) for 24 h. After that, cells were washed with prewarmed PBS three times. Cell nuclei and lysosomes were further stained with Hoechst 33342 (Beyotime, C1029), and Lyso-Tracker Red (Beyotime, C1046), respectively, followed by the observation under a confocal microscope. Changes in intracellular pH of ASFV-HN09 treated cells were assessed with 2′,7′-bis(2-carboxyethyl)-5(6)-carboxyfluorescein acetoxymethyl ester (BCECF-AM) (5 µmol/L, 30 min at 37°C). BCECF-AM is a membrane-permeant fluorescent indicator for the measurement of intracellular pH.

### Real-time RT-PCR

PAMs infected with ASFV were used for total RNA extraction using TRIzol (Invitrogen, 15596026). The RNAs were transcribed into cDNAs by FastKing gDNA Dispelling RT SuperMix (Tiangen, KR118-02). The transcriptional level of target gene mRNA was examined by a relative qRT-PCR assay using SYBR Premix Ex Taq II (TaKaRa, DRR081A), which was normalized to the transcription of a reference gene, the porcine *β-actin* gene. Relative fold changes in gene transcription were determined by the threshold cycle (2^−ΔΔCt^) method. The gene specific primers for RT-qPCR (Tsingke Biotechnology, China) used above were listed in Table 1.

**Table 1:** The RT-qPCR primers used in this study.

Primer name	Primer sequence (5′-3′)
*iNOS*	F: CATCACCACGCCTCCAACTCAG
	R: AGTCTCAAGCCTCTGCCTCTCG
*HO-1*	F: CAGGACATGGCCTTCTGGTA
	R: AGGGCCTTCTGAGCAATCTT
*NQO1*	F: GCTTACACATACGCTGCCAT
	R: GCCACAGAAATGCAAAGTGC
*SOD2*	F: TGAAGTTCAACGGTGGAGGC
	R: TCCTTGTTGAAACCGAGCCA
*ATP6V0A*	F: GCCTCACTCTATCCCTGTCC
	R: CCTTCATCTTCCGCACCTTG
*ATP6V0B*	F: CATCGGCCATCGAAACTACC
	R: ACTGCCAAAGATCTCCACGA
*ATP6V0C*	F: GCATCGCAGCCATGTCTGTCAT
	R: TGCCACCACCAGGCCATAGAT
*ATP6V0D1*	F: AACCATGCCTATGAGCCACT
	R: TCTGCTCAAAGCTGCCTAGT
*ATP6V0E*	F: ATCGTGATGAGCGTGTTCTG
	R: AGAGGAACTCTCTTCGTGACA
*LAMP1*	F: CCGACTCCAGTCTCGTGATTGC
	R: CGCTGTAACGGGTGGCATTTCT
*LAMP2*	F: CTCCTTATGCTCTGCCTCGT
	R: GTTGCAGCACCAAAGTCTGA
*CTSD*	F: CATCGGCCATCGAAACTACC
	R: ACTGCCAAAGATCTCCACGA
*CTSK*	F: CAAGGCAGCTAAGTGCAGAG
	R: AGACCGCGTGGTTGAGATTA
*CTSL*	F: TGAAGAAGGAAGGCGGAGAG
	R: TCCCTTCTTGTGCTTCTGGT
*CTSO*	F: GGCCAAGAAGATGAAATGGCA
	R: TCCGCACAATCCAGTATGGG
*CTSZ*	F: AGAATGCCACGTCATCCAGA
	R: GATGTAGGCCTGGTCCTTGT
*β-actin*	F: CTCCATCATGAAGTGCGACGT
	R: GTGATCTCCTTCTGCATCCTGTC

### Live-cell imaging

PAMs for live-cell imaging were grown on coverslip-bottomed dishes (Thermo Fisher, 150680) and infected with ASFV-GFP or ASFV-mCherry at indicated MOI. At indicated time points, the cells were imaged for time-lapse GFP/mCherry and DIC microscopy with a Nikon A1 confocal microscope. In either case, the cells were maintained on a heated 35 mm stage and incubator with the temperature set at 37°C, humidity, and CO_2_ control for live-cell imaging. Fresh RPMI-1640 supplemented with 2% FBS was perfused onto the dish throughout the experiment. The acquisition of images was every 6 min in our experiments. The experiments only indicating post-infection images are shown in figures. Simultaneous acquisition of EGFP/mCherry fluorescence emission and transmitted light was performed. Manipulation and subsequent analysis of acquired images were carried out with Nikon A1 Confocal Software.

### Transmission electron microscopy

PAMs were fixed with 4% PFA, 2% glutaraldehyde (GLA), 0.1 M phosphate buffer (pH 7.4) for 90 min at room temperature. The pellet was then enrobed in low melting point agarose and post-fixed in 1% osmium tetroxide in cacodylate buffer and en bloc stained with 1% uranyl acetate. Following dehydration with acetone, it was embedded in epoxy (TAAB 812 resin) according to standard procedures (Electron Microscopy Sciences, CAU). After polymerization, 80-nm-thick (ultrathin) sections were obtained and stained with uranyl acetate and lead citrate. For negative staining, the β-propiolactone (0.05%)-inactivated extracellular virions obtained by ultracentrifugation were resuspended in PBS, absorbed onto copper mesh, and stained with 2% sodium phosphotungstate (pH = 7.0). Images were collected using a HITACHI HT7700 electron microscope operating at 80.0 kV.

### Lysosome isolation, purification and proteomic analysis

Mock and ASFV-infected PAMs were subjected to isolation and purification of lysosome using a commercial lysosome isolation kit (Bestbio, BB-3603) according to the manufacturer’s instructions. The enrichment of lysosomal markers (e.g., LAMP1/2), endoplasmic reticulum markers (e.g., PDI), and mitochondrial markers (e.g., COX IV) was assessed by Western blot before and after lysosomal fractionation to confirm the purity of the isolated lysosome-enriched fractions. The isolated intact lysosomal fractions were sent to Hangzhou PTM BioLab, Inc. for proteomic analysis.

### Cell transfection

Cells grown to 60–90% confluence were transfected with the specified plasmids or siRNAs shown in the relevant figures using Lipofectamine LTX reagent or Lipofectamine RNAiMAX reagent according to the manufacturer’s instructions.

### Quantification and statistical analysis

For immunostaining, the cells or images were randomly selected for analysis. All the graphs and relevant statistical tests used in the work were created by GraphPad Prism version 6.00 (La Jolla, CA, USA). Statistical comparisons were made using the two-tailed unpaired Student’s t-test and the results are shown as mean ± SD. ns: no significant difference, *P* > 0.05; *P < 0.05; ** *P* < 0.01; *** *P* < 0.001; **** *P* < 0.0001. Error bars indicate means ± standard deviations (SD). Statistical parameters including the definitions, exact values of n, what n represents, and statistical significance are reported in the figures and corresponding figure Legends.

### Ethics approval

The animal experiments in this study were approved by the Laboratory Animal Ethical Committee of China Agricultural University with the approval number AW72903202-1-2. All animal experiments were performed according to the Chinese Regulations of Laboratory Animals—The Guidelines for the care of Laboratory Animals (Ministry of Science and Technology of the People’s Republic of China) and Laboratory Animal Requirements of Environment and Housing Facilities (National Laboratory Animal Standardization Technical Committee).

## Supporting information

S1 Fig Construction of a PAM bactericidal assay model. A-B. Design strategy and identification of recombinant *E. coli* strains Scale bar, 200 µm. C. OD_600nm_-CFU growth curves of recombinant *E. coli* strains. D. Optimization of bacterial inoculum MOI and incubation time for PAM infection. Scale bar, 10 µm.
(TIF)

S2 Fig ASFV infection induces oxidative stress. A. ROS levels in WSL-R4 cells infected with ASFV (MOI 0.5) for 24 h hpi. Scale bar, 100 µm. B. ROS levels in PAMs infected with ASFV (MOI 0.5) at 24 hpi. Scale bar, 100 µm. C. iNOS transcription levels in PAMs and NO expression levels in supernatant at different time points post-ASFV infection (MOI 0.5) (mean of three independent experiments ± SD). D-E. SOD enzyme activity and MDA level in WSL-R4 cells and PAMs infected with ASFV (MOI 0.5) at different time points, respectively (mean of three independent experiments ± SD). F. Transcription levels of antioxidant genes (*NQO1, HO-1, SOD2*) in PAMs infected with ASFV (MOI 0.5) at 24 hpi (mean of three independent experiments ± SD). G. Temporal changes in the expression levels of oxidative stress-related proteins in PAMs infected with ASFV (MOI 0.5) at different time points. The time points indicated in the figure represent the following procedure: after viral incubation for 1.5 h, the viral inoculum was removed and replaced with fresh medium. Cells were then collected at the designated post-infection time points. *P* values were calculated by a two-tailed unpaired t-test. *ns*, *P* > 0.05; ** *P* < 0.01.
(TIF)

S3 Fig ASFV infection induces mitochondrial ROS production. A. Mitochondrial superoxide production levels in WSL-R4 cells infected with ASFV at different MOIs for 24 hpi. Scale bar, 250 µm. B. Mitochondrial superoxide production levels in PAMs infected with ASFV at different MOIs for 24 hpi. Scale bar, 500 µm. C. Mitochondrial superoxide production levels in PAMs infected with ASFV (MOI 0.5) for 24 hpi. Scale bar, 20 µm. D. Changes in mitochondrial membrane potential in PAMs infected with ASFV (MOI 0.5) for 24 hpi. Scale bar, 20 µm.
(TIF)

S4 Fig ASFV infection inhibits phagosome-lysosome fusion in PAMs. A-D. Colocalization of *E. coli* with LAMP1, LAMP2, CTSD, and ATP6V0D in PAMs infected with ASFV (MOI 0.5) for 24 hpi, and corresponding colocalization analysis. Scale bar, 20 µm. E. Colocalization of latex beads with Lyso-Tracker in PAMs infected with ASFV (MOI 0.5) at different time points, and corresponding colocalization analysis. Scale bar, 250 µm.
(TIF)

S5 Fig The functional enrichment analysis results for differentially expressed proteins. Bubble plots of GO (Biological Process, Cellular Component, Molecular Function), protein-domain and KEGG-pathway enrichment for lysosomal differential proteins between ASFV-infected (MOI 0.5) and uninfected PAMs. *P* values were calculated using Fisher’s exact test. The y-axis represents pathway description information, and the x-axis represents the functional enrichment degree (Fold enrichment) after Log_2_ transformation, with larger values indicating higher enrichment degrees; the color of dots indicates the enrichment significance *P* value, with bluer colors representing stronger enrichment significance; the size of dots indicates the number of differentially expressed proteins in the KEGG pathway, with larger dots indicating more differentially expressed proteins.
(TIF)

S6 Fig The functional enrichment analysis results for differentially expressed proteins. Enrichment bar chart of GO (Biological Process, Cellular Component, Molecular Function), protein-domain and KEGG-pathway enrichment for lysosomal differential proteins between ASFV-infected (MOI 0.5) and uninfected PAMs. *P* values were calculated using Fisher’s exact test. The y-axis represents corresponding pathway description information, and the x-axis represents the -Log_10_ transformed enrichment significance *P* value, with larger values indicating stronger enrichment significance.
(TIF)

S7 Fig KEGG-pathway analyses of differentially expressed proteins. A-B. KEGG-pathway map highlighting enriched phagosomes and lysosomal differential proteins between ASFV-infected (MOI 0.5) and uninfected PAMs. Red fill indicates differentially up-regulated proteins; blue fill indicates differentially down-regulated proteins; yellow fill indicates nodes containing both differentially up-regulated and down-regulated proteins.
(TIF)

S8 Fig Functional classification of differentially expressed proteins identified by quantitative proteomics. A-C. COG/KOG annotation, KEGG pathway classification and GO level-2 analysis of lysosomal differential proteins between ASFV-infected (MOI 0.5, 24 hpi) and uninfected Mock group.
(TIF)

S9 Fig Functional classification of differentially expressed proteins identified by quantitative proteomics. A-C. COG/KOG annotation, KEGG pathway classification and GO level-2 analysis of lysosomal differential proteins between ASFV-infected PAMs (MOI 0.5, 24 hpi) plus *E. coli* challenge (MOI 10, 6 h) and uninfected Mock PAMs plus *E. coli* challenge (MOI 10, 6 h).
(TIF)

S10 Fig ASFV infection induces LMP. A. Live-cell imaging with acridine orange (AO) staining of ASFV-infected PAMs (MOI 0.5) for 24 h. Scale bar, 10 µm. B. Time-course live-cell imaging with AO staining of ASFV-infected PAMs (MOI 0.5) at different time points. Scale bar, 10 µm. C. LDH activity in culture supernatants of WSL-R4 and PAMs infected with ASFV (MOI 0.5) for 24 h (mean of four independent experiments ± SD). D. Live-cell imaging with AO staining of PAMs infected with ASFV (MOI 0.5) for 24 h in the presence of 10 mM NAC. *P* values were calculated by a two-tailed unpaired t-test. *ns*, *P* > 0.05; * *P* < 0.05; ** *P* < 0.01; *** *P* < 0.001; **** *P* < 0.0001.
(TIF)

S11 Fig ASFV-induced ROS can trigger LMP but does not affect cytosolic pH. A. Infection of ASFV-mCherry in PAMs in the presence of 10 mM NAC. Scale bar, 500 µm. B. Cytosolic pH detection in PAMs infected with ASFV (MOI 0.5) for 24 h in the presence of 10 mM NAC. Scale bar, 500 µm. C. Plate count results of PAMs infected with ASFV (MOI 0.5) for 24 h in the presence of 10 mM NAC, followed by phagocytosis of *E. coli* (MOI 10) or *S. suis* (MOI 10) for 6 h. D. Plate count results of PAMs infected with recombinant ASFV-mCherry or ASFV-WT (MOI 0.5) for 24 h, followed by phagocytosis of *E. coli* (MOI 10) for 6 h. Data were presented as mean ± SD from n = 3 independent experiments. *P* values were calculated by a two-tailed unpaired t-test. *ns*, *P* > 0.05; * *P* < 0.05; ** *P* < 0.01; *** *P* < 0.001; **** *P* < 0.0001.
(TIF)

S12 Fig Interference with pH-related proteins leads to cytoplasmic alkalinization and viral replication inhibition. A. Knockdown efficiency of siRNAs in PAMs and WSL-R4 cells. B. Cytosolic pH measured with BCECF-AM 24 h after siRNA transfection. Scale bar, 500 µm. C. Viral replication assessed 24 h post ASFV-GFP (MOI 0.5) infection of WSL-R4 cells 24 h after siRNA transfection. Scale bar, 500 µm. Data were presented as mean ± SD from n = 3 independent experiments. *P* values were calculated by a two-tailed unpaired t-test. *ns*, *P* > 0.05; * *P* < 0.05; ** *P* < 0.01; *** *P* < 0.001; **** *P* < 0.0001.
(TIF)

S13 Fig Interference with pH-related proteins resulted in lysosomal acidification inhibition. A. Lysosomal acidification in PAMs visualized with LysoTracker 24 h after siRNA transfection. Scale bar, 20 µm. B. Analysis of the mean fluorescence intensity of lysosome-specific staining in PAMs.
(TIF)

S1 Raw Gel Raw data from the Western blot images in the study. This file contains the uncropped, original Western blot membrane images for all experiments presented in the manuscript. The images are provided to verify the specificity and integrity of the antibody signals shown in the main figures.
(PDF)

S1 Data Combined raw data for all quantitative experiments. This Excel workbook contains the primary numerical data underlying all quantitative analyses.
(XLSX)

S2 Data Differentially expressed statistics. (XLSX)
