# Supplementary material for: African swine fever virus impairs porcine alveolar macrophages bactericidal function by disrupting lysosomal acidification and cathepsin activity
Source: PLoS Pathog. 2026 Sep 10;22(9):e1014573. doi: 10.1371/journal.ppat.1014573 (PMC13588506; doi:10.1371/journal.ppat.1014573)
Supplement: Supplementary file 1 [file ppat.1014573.s014.pdf]

chemiluminescence (ECL)

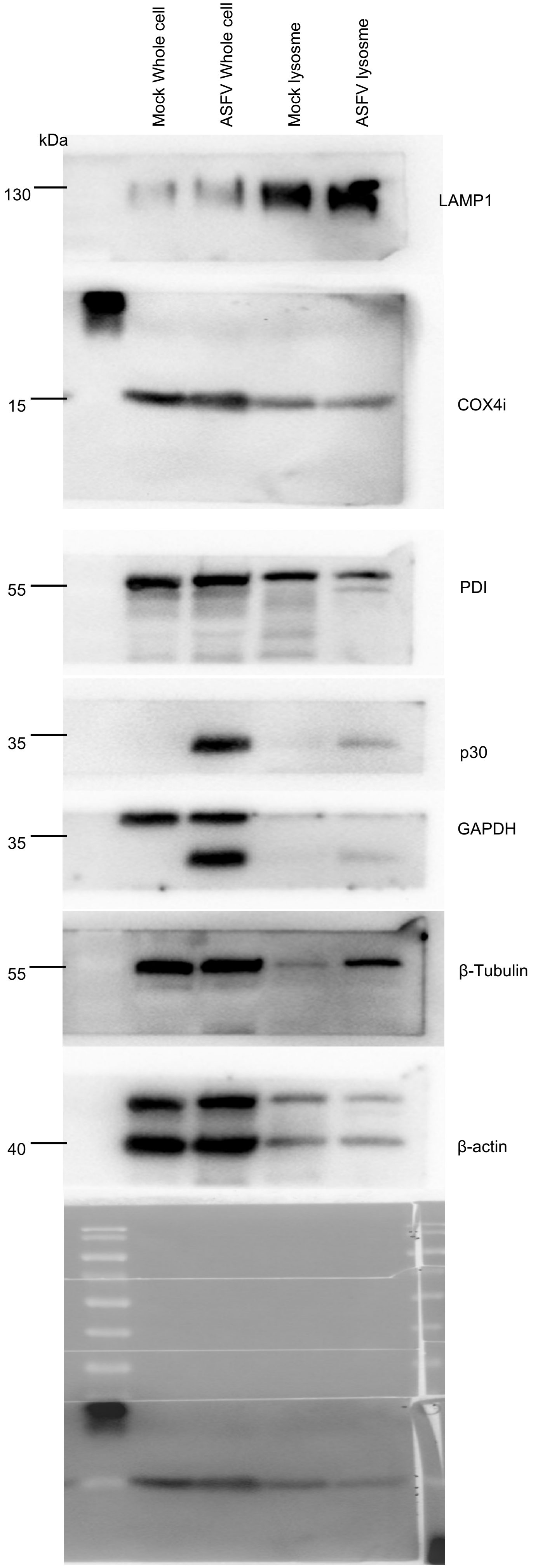

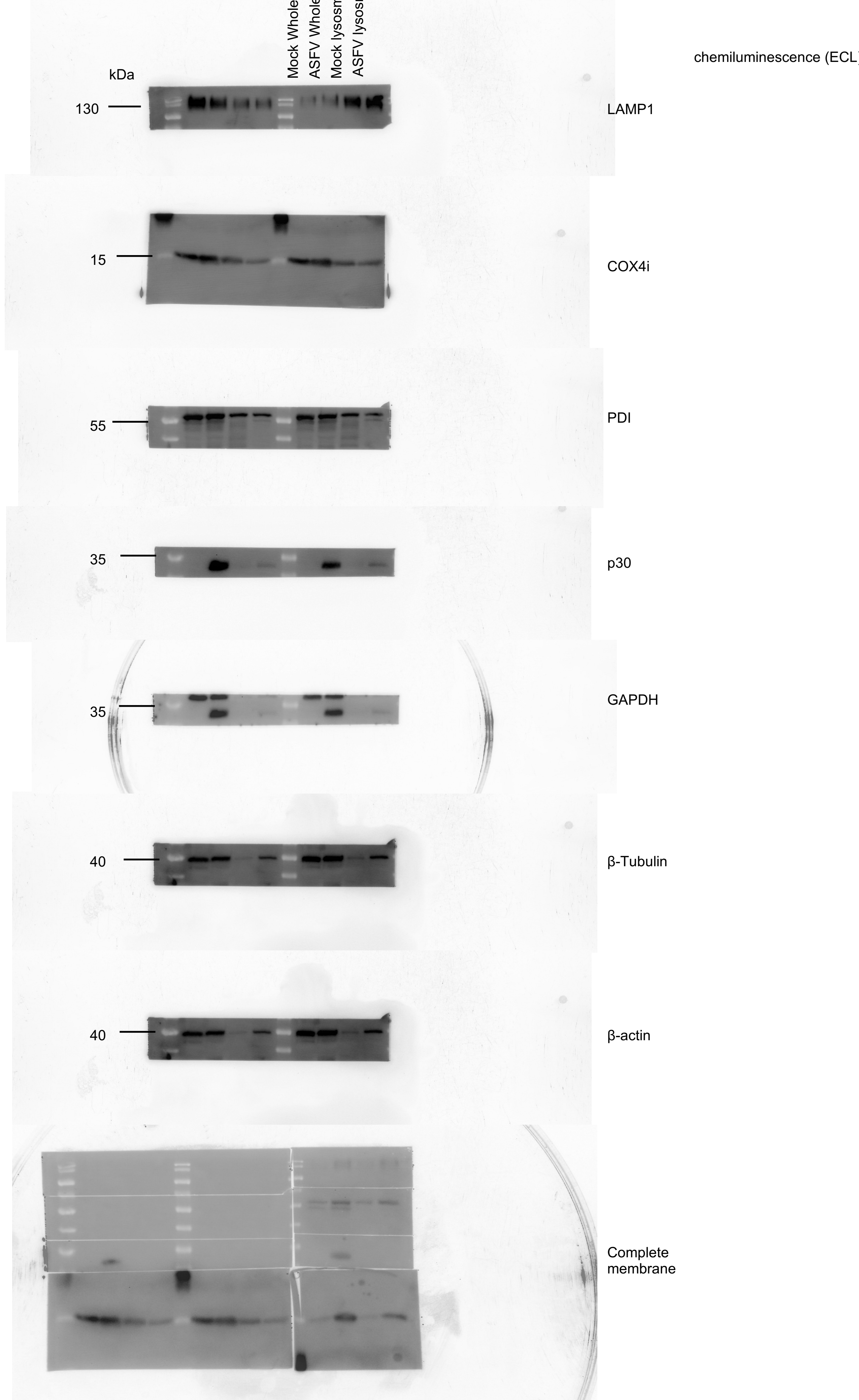
chemiluminescence (ECL)
Mock Whole
ASFV Whole
Mock lysosm
ASFV lysos
kDa
130
LAMP1
15
COX4i
55
PDI
35
p30
35
GAPDH
40
β-Tubulin
40
β-actin
Complete membrane

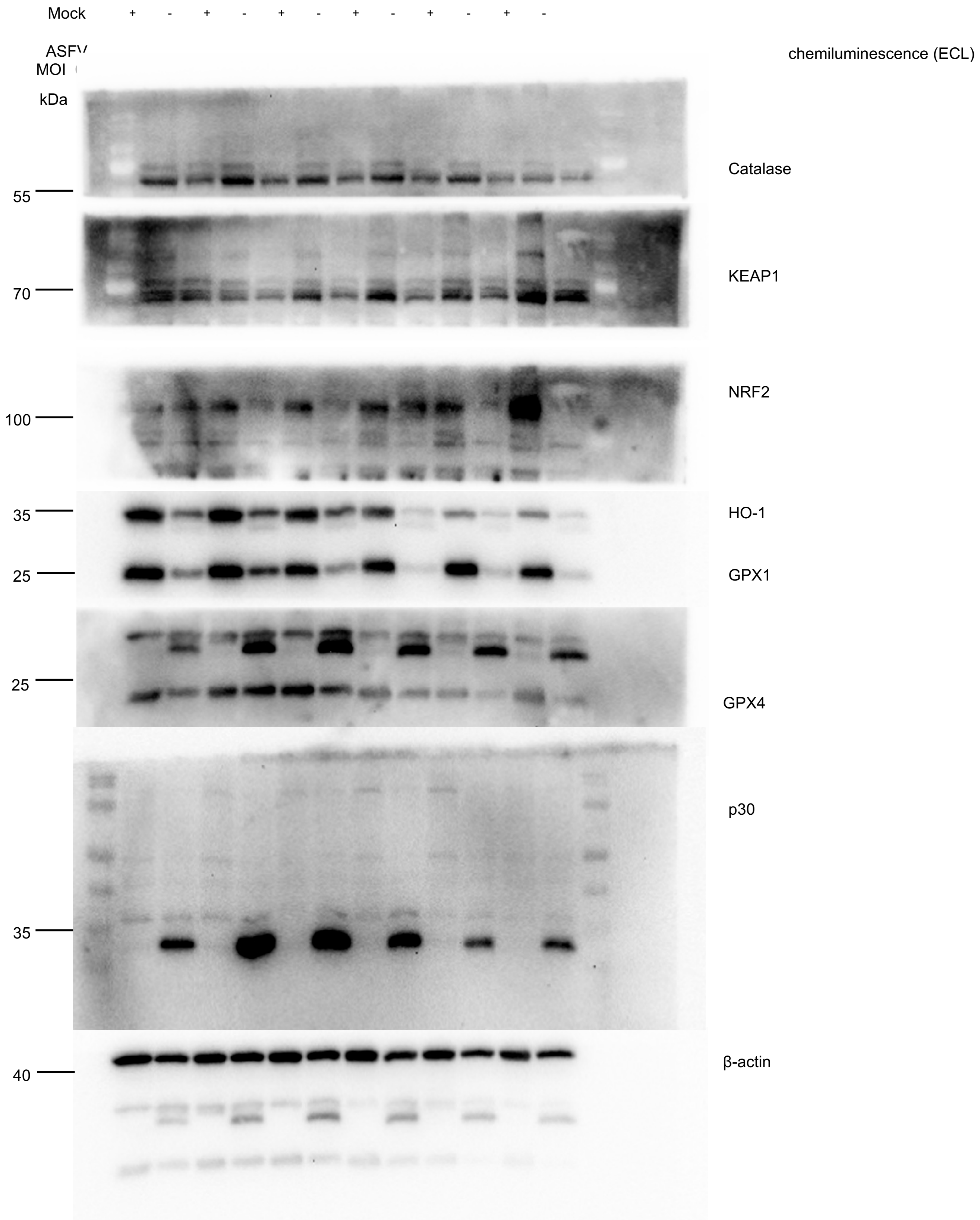
Mock + - + - + - + - + - + -
kDa
chemiluminescence (ECL)
55
Catalase
70
KEAP1
100
NRF2
35
HO-1
25
GPX1
25
GPX4
p30
35
40
β-actin

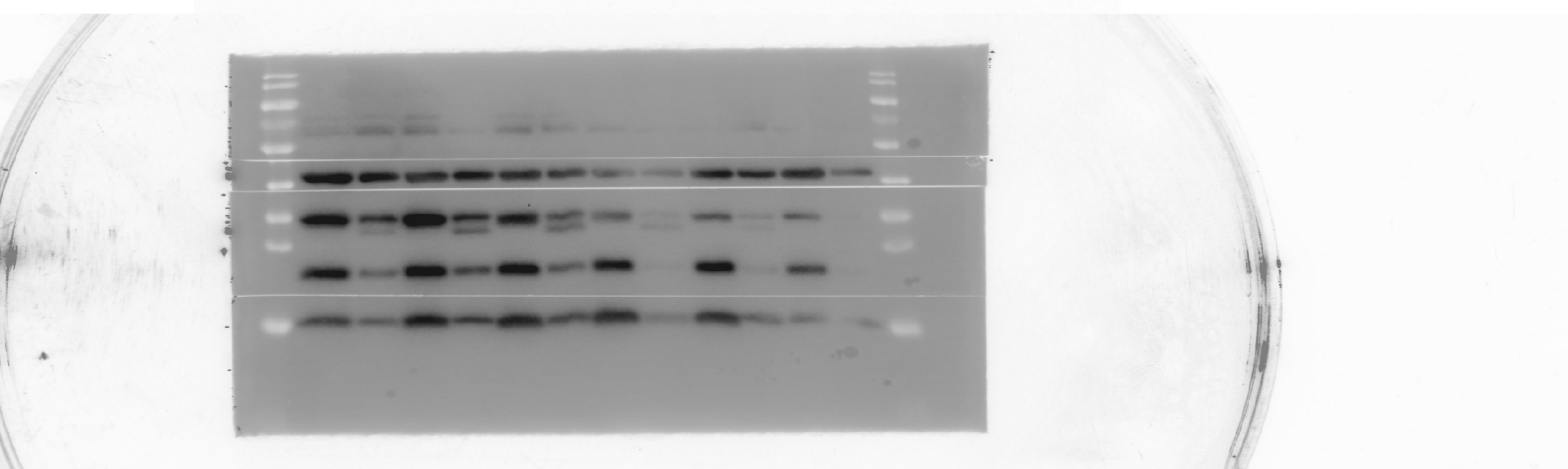

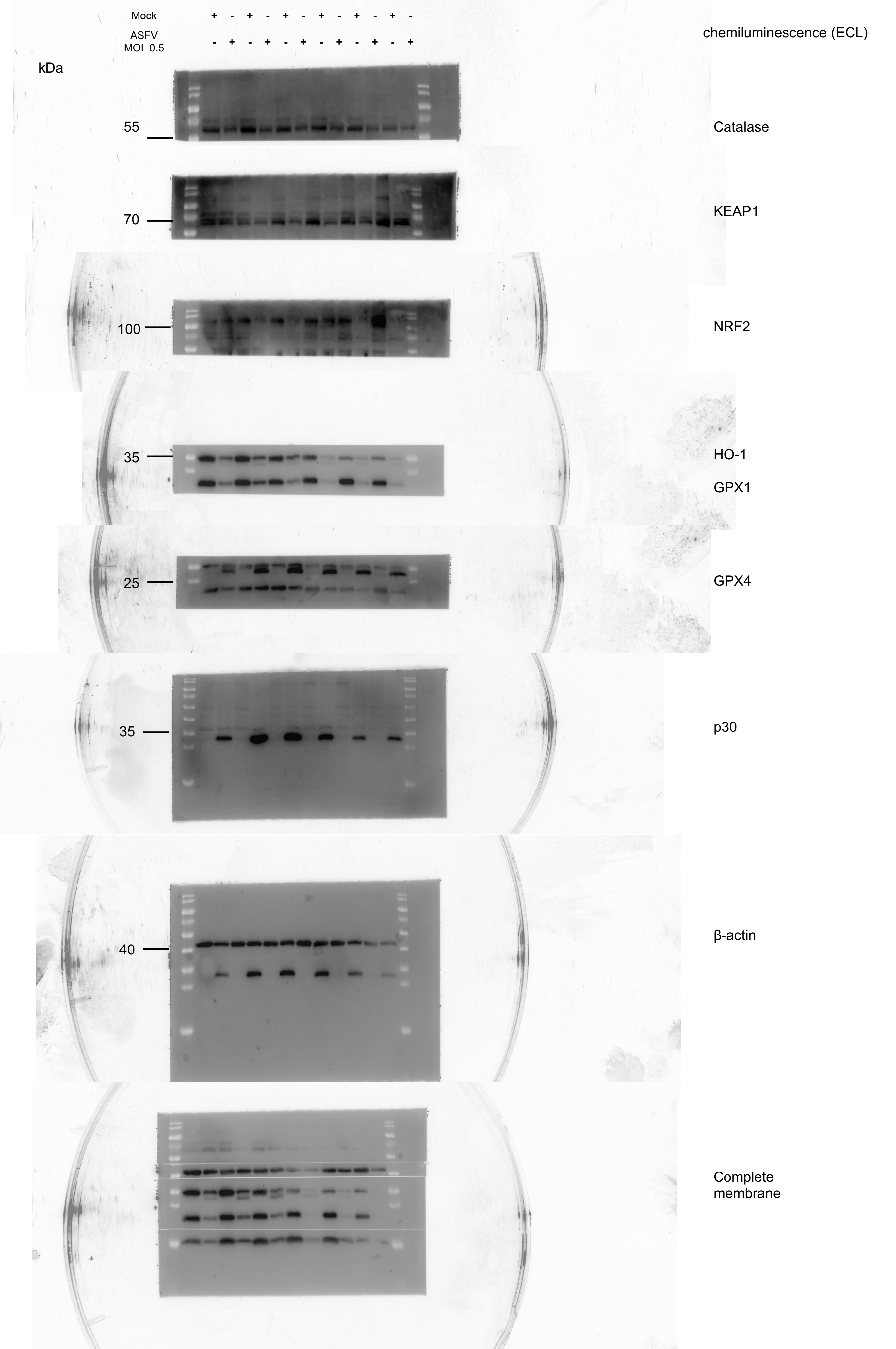
Mock + - + - + - + - + - + -
ASFV MOI 0.5 - + - + - + - + - + - +
kDa
chemiluminescence (ECL)
55
Catalase
70
KEAP1
100
NRF2
35
HO-1
GPX1
25
GPX4
35
p30
40
β-actin
Complete membrane
